# Epitranscriptomic Reprogramming Is Required to Prevent Stress and Damage from Acetaminophen

**DOI:** 10.3390/genes13030421

**Published:** 2022-02-25

**Authors:** Sara Evke, Qishan Lin, Juan Andres Melendez, Thomas John Begley

**Affiliations:** 1 Nanobioscience Constellation, College of Nanoscale Science and Engineering, SUNY Polytechnic Institute, Albany, NY 12203, USA; sevke@sarepta.com (S.E.); jmelendez@sunypoly.edu (J.A.M.); 2 The RNA Institute, University at Albany, Albany, NY 12222, USA; qlin@albany.edu; 3 Department of Biological Sciences, University at Albany, Albany, NY 12222, USA; 4 RNA Epitranscriptomics and Proteomics Resource, University at Albany, Albany, NY 12222, USA

**Keywords:** RNA modification, stress response, acetaminophen, Alkbh8, epitranscriptomic, tRNA

## Abstract

Epitranscriptomic marks, in the form of enzyme catalyzed RNA modifications, play important gene regulatory roles in response to environmental and physiological conditions. However, little is known with respect to how acute toxic doses of pharmaceuticals influence the epitranscriptome. Here we define how acetaminophen (APAP) induces epitranscriptomic reprogramming and how the writer Alkylation Repair Homolog 8 (Alkbh8) plays a key gene regulatory role in the response. Alkbh8 modifies tRNA selenocysteine (tRNA^Sec^) to translationally regulate the production of glutathione peroxidases (Gpx’s) and other selenoproteins, with Gpx enzymes known to play protective roles during APAP toxicity. We demonstrate that APAP increases toxicity and markers of damage, and decreases selenoprotein levels in *Alkbh8* deficient mouse livers, when compared to wildtype. APAP also promotes large scale reprogramming of many RNA marks comprising the liver tRNA epitranscriptome including: 5-methoxycarbonylmethyluridine (mcm^5^U), isopentenyladenosine (i^6^A), pseudouridine (Ψ), and 1-methyladenosine (m^1^A) modifications linked to tRNA^Sec^ and many other tRNA’s. Alkbh8 deficiency also leads to wide-spread epitranscriptomic dysregulation in response to APAP, demonstrating that a single writer defect can promote downstream changes to a large spectrum of RNA modifications. Our study highlights the importance of RNA modifications and translational responses to APAP, identifies writers as key modulators of stress responses in vivo and supports the idea that the epitranscriptome may play important roles in responses to pharmaceuticals.

## 1. Introduction

Epitranscriptomic marks in the form of enzyme-catalyzed RNA modifications are important gene regulatory signals, with defects linked to disrupted gene expression, disease onset and disease progression [1]. Modifications on mRNA, rRNA, and tRNA have been shown to be dynamically regulated to allow cells to survive different stressors and adapt to changes in cellular physiology [2,3,4,5,6,7,8,9]. In addition, the location and levels of specific RNA modifications have been shown to drive gene expression. tRNAs are the most heavily modified RNA species, and RNA modifications are critical for regulating tRNA structure, function, and stability. Writer enzymes can catalyze or add tRNA modifications, while eraser enzymes can remove modifications and reader enzymes bind and recognize modifications. Chemical modifications on tRNAs provide regulation of structure and function, while those occurring in the anticodon loop positions 34 to 37 can regulate translation and fidelity. The wobble position (34) in the anticodon stem loop of tRNA allows pairing to occur with more than one nucleoside, allowing for a single tRNA to decode multiple codons with different 3′ nucleosides [10,11,12]. Wobble position modifications to uridine (U), cytidine (C), guanosine (G), and adenosine (A) have all been reported and can include simple methylations to more elaborate chemical additions, which can regulate anticodon-codon interactions while preventing translational errors [10,11].

While most mammalian tRNAs have 3 to 17 positions modified [13], the tRNA for selenocysteine (tRNA^Sec^) is unique because it only has 4 positions modified. tRNA^Sec^ modifications and writers include the 5-methoxycarbonylmethyluridine (mcm^5^U) and the ribose-methylated derivatived, 5-methoxycarbonylmethyl-2′-O-methyluridine (mcm^5^Um) at position U34 which are dependent on alkylation repair homolog 8 (Alkbh8) along with its accessory protein tRNA methyltransferase 112 (Trm112). The isopentenyladenosine (i^6^A) modification at position A37 is catalyzed by tRNA isopentenyltransferase 1 (Trit1) [14]. The pseudouridine (Ψ) modification at position U55 is catalyzed by pseudouridine synthase 4 (Pus4) also known as TruB pseudouridine synthase family member 1 (Trub1) [15]. The 1-methyladenosine (m^1^A) modification at position A58 is catalyzed by the catalytic subunit of tRNA (adenine-N_1_-)-methyltransferase 61A (Trmt61A) and RNA binding protein non-catalytic subunit tRNA (adenine (58)-N(1))-methyltransferase 6 (Trm6) [16]. Alkbh8, mcm^5^U and mcm^5^Um have been shown to play a critical role in the translation of selenoproteins [17,18,19,20,21]. Selenoprotein synthesis is orchestrated by interactions between cis RNA elements and trans proteins and specifically modified tRNA to recode a UGA stop codon. Thus, the UGA selenocysteine (Sec) codon found within selenoprotein genes works with Alkbh8-modified tRNA, as well as other factors, to promote the translation of selenoproteins. There are 25 selenoproteins in human systems with rodents having 24, with glutathione peroxidase 6 (Gpx6) being the discrepant selenoprotein [22]. Some examples of selenoproteins include glutathione peroxidase (Gpx)1-4, thioredoxin reductases (TrxR) 1-3, selenoprotein S (SelS), selenoprotein K (Selk), and selenoprotein P (Selp).

Selenoproteins play roles in stress responses, regulating metabolism, immunity, and in embryonic vitality and development [23,24,25,26,27,28,29]. Many selenoproteins serve as antioxidant enzymes to mitigate damage caused by reactive oxygen species (ROS) [29,30,31,32,33,34,35,36,37]. A GeneTrap generated mouse deficient in *Alkbh8* (*Alkbh8^Def^*) has been used to generate embryonic fibroblasts (MEFs), and these cells have been used to demonstrate that writer defects promote increased levels of intracellular ROS and DNA damage and reduced selenoprotein expression [17]. *Alkbh8^Def^* MEFs also display a proliferative defect, accelerate senescence and fail to increase selenoprotein levels in response to hydrogen peroxide (H_2_O_2_) [17,38]. *Alkbh8^Def^* mice also have increased lung inflammation and a disruption in lung glutathione levels [36]. Pulmonary exposure of *Alkbh8^Def^* mice to naphthalene, the common polyaromatic hydrocarbon found in mothballs, increased stress markers and lung damage, when compared to their wildtype (WT) counterparts [36]. Overall, these findings demonstrate that the epitranscriptomic writer *Alkbh8* plays a vital role in protection from environmental stressors.

Little is known with respect to how the epitranscriptome responds to pharmaceutical stress. Acetaminophen (APAP) is one of the most prevalent over the counter drugs due to its analgesic and antipyretic properties and is consumed regularly by over 60 million Americans each week. APAP is a safe and effective drug that combats various ailments. However, in the United States nearly 50% of all cases of acute liver failure are due to an excessive use of APAP [39]. Gpx proteins have been linked to preventing APAP toxicity by providing protection from its metabolized forms [40]. The majority of APAP (85–90%) is acted upon by phase II metabolism where APAP is chemically altered by UDP-glucuronosyl transferases (Ugt) and sulfotransferases (Sult), and then converted to glucuronidated and sulfated metabolites that are eliminated in urine. A small amount (~2%) of APAP is excreted in urine without modification and the remaining is biometabolized by the cytochrome enzyme, Cyp2e1, which results in the highly reactive, toxic metabolite *N*-acetyl-para-benzoquinone imine (NAPQI) [41]. Sufficient glutathione (GSH) in hepatocytes will reduce the reactive NAPQI and promote excretion in the urine. However excessive amounts of NAPQI can increase oxidative stress, decrease GSH, and promote mitochondrial dysfunction leading to depletion of adenosine triphosphate (ATP) stores [42].

Selenoproteins have been shown to provide protection from APAP toxicity. Mirochnitchenko et al. demonstrated that glutathione peroxidase injection provided complete protection in mice administered an acute toxic dose of APAP [40]. GPX3 overexpressing cells are more resistant to APAP toxicity than cells whose GPX3 levels were decreased by siRNA [43]. Human GPX and mouse Gpx proteins are clearly linked to preventing APAP toxicity. As selenoprotein synthesis and activity are reliant on RNA modifications that drive selenocysteine utilization, we hypothesized that Alkbh8-regulated epitranscriptomic marks would be involved in the response to APAP. Here we test the hypothesis that the epitranscriptomic writer, Alkbh8, protects against APAP toxicity by catalyzing tRNA modifications that regulate the translation of stress response genes. We have phenotypically characterized liver tissue from both C57BL/6 WT and *Alkbh8^Def^* treated mice with either saline (vehicle control) or APAP under both acute (6 h) and sub-chronic (4 daily injections) exposure conditions. When challenged with APAP, WT liver tissue showed an increase in many selenoproteins whereas the *Alkbh8^Def^* liver tissues were disrupted in their expression and show classic hallmarks of stress and damage, including increased expression of the oxidative stress marker, 8-isoprostane, and liver damage marker, alanine transaminase (ALT). Transcriptional responses to acute APAP were similar between the genotypes; however, protein level differences were detected, which is indicative of post-transcriptional changes and translational regulation reflective of epitranscriptomic regulation. After a single dose of APAP, we have discovered that there is significant change in the levels of many tRNA modifications in response to APAP. Increases in the levels of tRNA modifications specific to tRNA^Sec^ and many other tRNA isoacceptors were identified, with dysregulation of the many APAP dependent marks observed in *Alkbh8^Def^* mice. Our study is one of the first to characterize the roles of epitranscriptomic marks and writers in response to APAP, and supports the idea that the epitranscriptome adapts to and modifies the effects of pharmaceuticals.

## 2. Materials and Methods

### 2.1. Animal Experiments

Animal studies were carried out in strict accordance with recommendations in the guide for the Care and Use of Laboratory Animals of the National Institutes of Health. The protocol was approved by the University at Albany Institutional Animal Care and Use Committee (Albany, NY, USA), protocol #17-016/#20-014 for breeding and #18-010 for APAP exposure. Two homozygous strains of mice of the C57BL/6J background were bred, WT and *Alkbh8^Def^*. Wild-type mice were purchased from Taconic Biosciences (Rensselaer, NY, USA). *Alkbh8^Def^* were produced through an insertional mutagenesis approach targeting the *Alkbh8* gene in the parental E14Tg2a.4 129P2 embryonic stem cell line, as previously described [17]. *Alkbh8^Def^* mice were produced using a vector containing a splice acceptor sequence upstream of a β-geo cassette (β-galactosidase/neomycin phosphotransferase fusion), which was inserted into intron 7 at chromosome position 9:3349468-3359589, creating a fusion transcript of Alkbh8-β-geo [44]. Multiplex qRT-PCR and relative cycle threshold analysis (ΔΔCT) on genomic DNA derived from tail biopsies were used to determine animal zygosity for *Alkbh8^Def^*, using TaqMan oligonucleosides specific for neomycin (Neo, target allele) and T-cell receptor delta (Tcrd, endogenous control). Neo forward (5′-CCA TTC GAC CAC CAA GCG-3′), Neo reverse p (5′-AAG ACC GGC TTC CAT CCG-3′), Neo Probe (5′-FAM AAC ATC GCA TCG AGC GAG CAC GT TAMRA-3′), Tcrd forward (5′-CAG ACT GGT TAT CTG CAA AGC AA-3′), Tcrd reverse (5′-TCT ATG CAA GTT CCA AAA AAC ATC-3′), and Tcrd probe (5′-VIC ATT ATA ACG TGC TCC TGG GAC ACC C TAMRA-3′) primers were used for genotyping.

For all animal studies, male mice between 8–12 weeks of age were used. Females were not used, as they have been shown to be less susceptible to APAP liver injury due to their accelerated recovery of hepatic glutathione (GSH) levels [45]. Mice were sacrificed via CO_2_ asphyxiation and in some cases, followed by non-survival vena cava puncture and necropsy of the liver. Whole blood was allowed to clot for 30 min at 25 °C, and organs were flash-frozen and placed in −80 °C for storage.

### 2.2. APAP Solution Preparation and Intraperitoneal Injection (I.P.)

APAP and Dulbecco’s phosphate buffered saline (PBS) were purchased from Sigma-Aldrich (St. Louis, MO, USA). A 5 mg/mL (*w*/*v*) solution of APAP was made in a 15 mL conical tube with 100 mL of PBS and 0.5 g of APAP, due to lower solubility property of APAP, the solution was boiled until dissolved and then cooled to room temperature before injection. Solutions were made fresh each day. For intraperitoneal injections (I.P.) mice were first weighed and then placed under 5% isoflurane gas for approximately three minutes. The mice were then manually restrained, the injection site was wiped with 70% ethanol, and either saline or 600 mg/kg APAP solution was injected into the intraperitoneal cavity in the lower left abdominal quadrant using Coviden™ Monoject™ 27g, ½ inch standard hypodermic needles (Fisher Scientific, Franklin, MA, USA) and sterile 1 mL Coviden™ Monoject™ tuberculin syringes (Fisher Scientific, Franklin, MA, USA). Injections for acute exposures occurred once and six hours after the exposure mice were sacrificed. For chronic conditions, injections were administered at the same time daily for four days, every 24 h, and mice were sacrificed 24 h post last injection. After injection, mice were observed up to one hour for recovery from anesthesia and to ensure mice were not experiencing any pain or distress. Mice were further monitored for signs of excess stress and weight loss, with any mouse losing more than 20% of its body weight sacrificed based on IACUC guidelines.

### 2.3. Alanine Transaminase (ALT) Assay

The Alanine Transaminase Colorimetric Activity Assay (Catalog no. 700260, Cayman Chemical, Ann Arbor, MI, USA) was performed following manufacturer’s protocol to provide an indicator of liver damage. WT and *Alkbh8^Def^* were exposed to either saline or APAP as described above and sacrificed via CO_2_ asphyxiation. Cardiac puncture was then performed using Coviden™ Monoject™ 27g, ½ inch standard hypodermic needles (Fisher Scientific, Hampton, NH, USA) and sterile 1 mL Coviden™ Monoject™ tuberculin syringes (Fisher Scientific, Hampton, NH, USA) to collect a minimum volume of 200 µL of blood. Serum from each sample was prepared by allowing the blood to clot at 25 °C for 30 min. Samples were centrifuged at 2000× *g* for 15 min at 4 °C and the top serum layer was collected and stored at −80 °C. Serum samples were processed based on Alanine Transaminase Colorimetric Activity Assay Kit (catalog no. 700260, Cayman Chemical, Ann Arbor, MI, USA) instructions, and absorbance was monitored at 340 nm over a period of 10 min at 1-min intervals. Data analysis was performed, and ALT activity was reported as U/L. Statistical significance was calculated utilizing three biological replicates for each exposure condition and genotype, with error bars denoting standard error of the mean and significance determined using Student’s unpaired *t*-test in GraphPad Prism version 9.0 (GraphPad, San Diego, CA, USA).

### 2.4. 8-Isoprostane Assay

The 8-isoprostane assay (Catalog no. ab175819, Abcam, Cambridge, MA, USA) was performed following manufacturer’s protocol. Mice were exposed to either saline or APAP as described above and sacrificed via CO_2_ asphyxiation. Then, 1X phosphate buffered saline (PBS) was injected into the portal vein and hepatic artery, to remove excess blood and other cellular debris from the liver. Once dissected from the mouse, the livers were washed in PBS and then stored at −80 °C. Additional reagents that were used included 2N Sulfuric Acid Stop Solution (Catalog no. DY994, R&D Systems, Minneapolis, MN, USA), triphenylphosphine (TPP) (Catalog no. T84409-1G, Sigma-Aldrich, St. Louis, MO, USA), and ethyl acetate (Catalog no. 270989, Sigma-Aldrich, St. Louis, MO, USA). 8-isoprostane was measured following manufacturer’s protocol with the adjustment for low sample volume. Livers were weighed and homogenized in a 500 µL solution containing 5 mg TPP in dH_2_O, and then acidified by adding 4 µL of glacial acetic acid to ensure pH was ~4.0. Next, 500 µL of ethyl acetate was added to each sample, vortexed and centrifuged at 5000 rpm for 5 min to separate the organic phase. The supernatant was collected and placed in a new conical tube and the previous step was performed one more time. The pooled organic phase supernatant was dried using nitrogen gas and then reconstituted in 20 µL 100% ethanol. Samples were further processed according to manufacturer’s protocol and then used for ELISA measurement and analyzed. Statistical significance was calculated utilizing three biological replicates for each exposure condition and genotype with error bars denoting standard error of the mean and significance determined using Student’s unpaired *t*-test in GraphPad Prism v9.

### 2.5. Protein Quantification Assays

Primary antibodies used for quantification were as follows: TrxR1 (catalog no. MAB7428, R&D Systems, Minneapolis, MN, USA), TrxR2 (catalog no. ab180493, Abcam, Cambridge, MA, USA), Gpx1 (Catalog no. AF3798, R&D Systems, Minneapolis, MN, USA), Gpx3 (Catalog #AF4199, R&D Systems, Minneapolis, MN, USA), Gpx4 (catalog no. 565320, Novus Biologicals, Centennial, CO, USA), SelS (catalog no. HPA010025, Sigma-Aldrich, St. Louis, MO, USA), and Gapdh (catalog no. CB1001, Millipore Sigma, Burlington, MA, USA). Liver tissue was homogenized in 1 mL RIPA Buffer (50 mM Tris-HCl, pH 7.4, with 150 mM NaCl, 1% TridonX-100, 0.5% sodium deoxycholate, and 0.1% sodium dodecyl sulfate) supplemented with an EDTA-free phosphatase inhibitor cocktail 2 (catalog no. P5726, Sigma-Aldrich, St. Louis, MO, USA) and phosphatase inhibitor cocktail 3 (catalog no. P0044, Sigma-Aldrich, St. Louis, MO, USA). Livers were agitated periodically on ice for 30 min and then spun at 15,000× *g* for 12 min at 4 °C. The supernatant was then collected, and protein levels were quantified via Bradford assay (BioRad, Portland, OR, USA) according to manufacturer’s instructions at a monochromatic wavelength of 595 nm. Proteins were then normalized to 1.5 µg/mL concentration and added to Wes (ProteinSimple, San Jose, CA, USA) protein analysis plates (catalog no.SM-W004, ProteinSimple, San Jose, CA, USA) and quantified with Compass for Simple Western v2.1. Primary antibodies of interest were always multiplexed with endogenous Gapdh primary antibody to normalize chemiluminescence area under the curve. Statistical significance was calculated utilizing three biological replicates for each exposure condition and genotype, error bars denote standard error mean with statistical significance determined using Student’s unpaired *t*-test in GraphPad Prism v9.

### 2.6. Liquid Chromatography-Tandem Mass Spectrometry (LC-MS/MS)

Small RNAs were first isolated from liver tissue using PureLink™ miRNA Isolation Kit (catalog no. K157001, Invitrogen, Waltham, MA, USA). RNA concentration was then quantified at The Center for Functional Genomics/University at Albany using an Agilent Technologies 2100 Bioanalyzer System (Santa Clara, CA, USA) and the respective Bioanalyzer Small RNA reagents (catalog no. 5067-1548, Agilent Technologies, Santa Clara, CA, USA). Purified small RNA samples were then digested using Nucleoside Digestion Mix (catalog no. M0649S, New England BioLabs, Beverly, MA, USA). LC-MS/MS measurement of various post-transcriptional modifications were performed on a Waters XEVO TQ-S^TM^ (Waters, Milford, MA, USA) electrospray triple quadrupole mass spectrometer configured with ACQUITY I class ultra-performance liquid chromatography, as previously described [46]. The capillary voltage was set at 1.0 kV with extraction cone of 14 V. Nitrogen flow was maintained at 1000 L/h and desolvation temperature at 500 °C. The cone gas flow was set to 150 L/h and nebulizer pressure to 7 bars. Each individual nucleoside modification was characterized by single infusion in a positive mode ionization over an *m*/*z* range of 100–500 amu. Further nucleoside characterization was performed using Waters software part of Intellistart MS/MS method development program where a ramp of collision and cone voltages is applied to find optimal collision energy parameters for the daughter ions. To quantify various RNA modified nucleosides, calibration curves were prepared for these modified nucleosides including adenosine, cytidine, guanosine, and uridine. Stable isotope labelled guanosine [^13^C][^15^N]-G (1 pg/µL) (cat# CNLM-3808-CA-5, Cambridge Isotope Laboratories, Inc. Tewksbury, MA, USA) was purchased and used as an internal standard.

A method to extract peak areas from raw data to allow quantification was developed using a combination of instrument manufactures’ suites, MassLynx V4.1 and TargetLynx (Waters, Milford, MA, USA). These methods allowed extraction of information to produce calibration curves from each RNA modification standard. In addition, these programs were used to extract the peak areas to be extrapolated on the standard calibration curves for quantification of RNA modifications. In house Python scripts combined with Originlab software suite 2017 (OriginLab Corporation, Northampton, MA, USA) was used to quantify various RNA modifications. Heat maps were generated using MORPHEUS versatile matrix visualization and analysis software (https://software.broadinstitute.org/morpheus, accessed on 10 January 2021).

### 2.7. RNA Next-Generation Sequencing

Total RNA was isolated from liver tissue. Samples were placed in 1 mL of Trizol reagent (Invitrogen, Waltham, CA, USA), then homogenized with a Tissue-Tearor homogenizer (BioSpec, Bartlesville, OK, USA). RNA isolation was performed using manufacturer’s protocol (catalog no. 15596026, Thermo Fisher Scientific, Waltham. MA, USA). Isolated RNA samples were treated with DNase I, RNase-free (1 U/µL) (catalog no. EN0521, Thermo Fisher Scientific, Waltham. MA, USA) following manufacturer’s instruction. Samples were then processed at The Center of Functional Genomics/University at Albany for differential expression with Poly-A selection and sequenced using an Illumina Nextseq500 (Illumina, Inc., San Diego, CA, USA) at 75 million single-end reads per run. FASTQ files are available on NCBI Gene Expression Omnibus (GSE169155). Analysis was performed through bash and differential expression was analyzed using R Studio v1.3.1093 (Team 2020) (Boston, MA, USA). Enhanced volcano plots were created using DESeq2 package [47] and EnhancedVolcano with a fold change (FC) cutoff of 1.0 and a pCutoff of 0.05. Gene count plots of epitranscriptomic writers were created using DeSeq2 and ggplot2 packages. Metascape analysis [48] was performed with upregulated genes of a log_2_fold change cut-off of 2.0 or greater and ensembl ID lists were uploaded, respectively, per comparison condition. Statistical significance was calculated utilizing three biological replicates for each exposure condition and genotype, and was determined using student’s unpaired *t*-test.

## 3. Results

### 3.1. Alkbh8^Def^ Mice Show Sensitivity to APAP Toxicity and Dysregulated Selenoprotein Expression in the Liver

**Figure 1:**
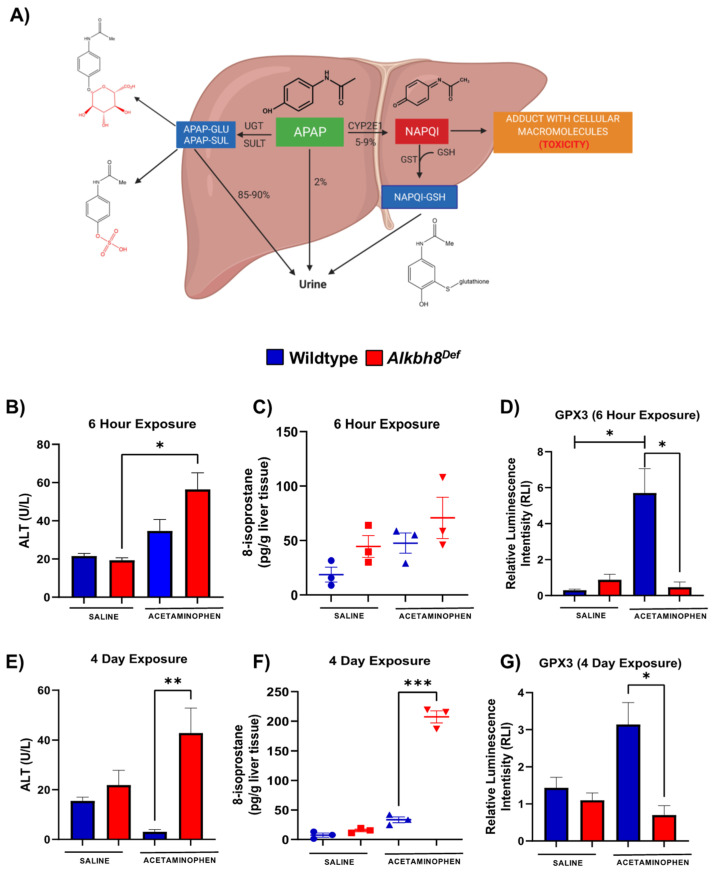
Writer deficient mouse livers have increased sensitivity to APAP induced stress and display decreased selenoprotein levels. (**A**) APAP can be converted into a non-toxic form by UDP-glucuronosyltransferases (UGT) or a toxic form by cytochrome P450 enzyme (CYP2E1) to generate the reactive intermediate metabolite N-actyl-p-benzoquinone imine (NAPQI). Male C57Bl/6 WT (blue) and *Alkbh8^Def^* (red) mice (8–12 weeks old) were exposed to a single dose of APAP (**B**–**D**) or daily dose of APAP over 4 days (**E**–**G**), and tissue/blood was harvested. (**B**,**E**) ALT and (**C**,**F**) 8-isoprostane levels were determined in blood and liver samples, respectively for WT saline (blue circle), *Alkbh8^Def^* saline (red square), WT APAP (blue triangle) and *Alkbh8^Def^* APAP (red triangle) (**D**,**G**) Gpx3 protein levels in the liver were evaluated using the ProteinSimple WES system. Statistical significance (N = 3) was measured by an unpaired *t*-test with (* *p* < 0.05, ** *p* < 0.01, and *** *p* < 0.001).

APAP can be bioactivated by a cytochrome P450 enzyme, Cyp2e1, to catalyze the formation of the reactive metabolite NAPQI (Figure 1A). The response to toxic levels of APAP (600 mg/kg) was studied in 8–12-week-old C57BL/6 male WT and *Alkbh8^Def^* mice. Stress biomarkers indicative of liver damage and oxidative stress were measured in serum derived from whole blood and liver tissue, respectively. Alanine aminotransferase (ALT) levels were significantly higher in *Alkbh8^Def^* livers 6 h after injection of APAP, relative to APAP exposed WT livers (Figure 1B). The lipid peroxidation product 8-isoprostane is a in vivo biomarker of oxidative stress [49]. For both saline and 6 h APAP exposure conditions for *Alkbh8^Def^* liver tissue there was a slight increase in 8-isoprostane levels compared to WT samples (Figure 1C), but it did not reach a significant level (*p* = 0.33). Glutathione peroxidase 3 (Gpx3) is an extracellular antioxidant enzyme that aids in scavenging hydrogen peroxide as well as other hydroperoxides [50]. In response to APAP, there is a significant increase (*p* = 0.02) in Gpx3 expression in WT compared to its saline control, and this increased response was not observed in *Alkbh8^Def^* liver tissue (Figure 1D).

Daily injections over 4 days were used to study chronic APAP use in the WT and *Alkbh8^Def^* mice. ALT (*p* = 0.004) and 8-isprostane levels (*p* = 0.0001) were measured and found to be significantly increased in the *Alkbh8^Def^* mice after APAP injection compared to WT APAP (Figure 1E,F). Gpx3 expression levels in WT and *Alkbh8^Def^* livers were also significantly (*p* = 0.02) different after 4 days of APAP exposure (Figure 1G). Similar to 6 h data, there was an APAP-induced increase in Gpx3 in WT livers, which was not observed in the *Alkbh8^Def^* mice. Our ALT, 8-isoprostane and Gpx3 data support the idea that writer deficient livers are experiencing APAP induced stress and defective selenoprotein synthesis.

### 3.2. Next-Generation Sequencing Analysis Reveals Similar Transcriptional Responses to a Single Dose of APAP between WT and Alkbh8^Def^ Livers

**Figure 2:**
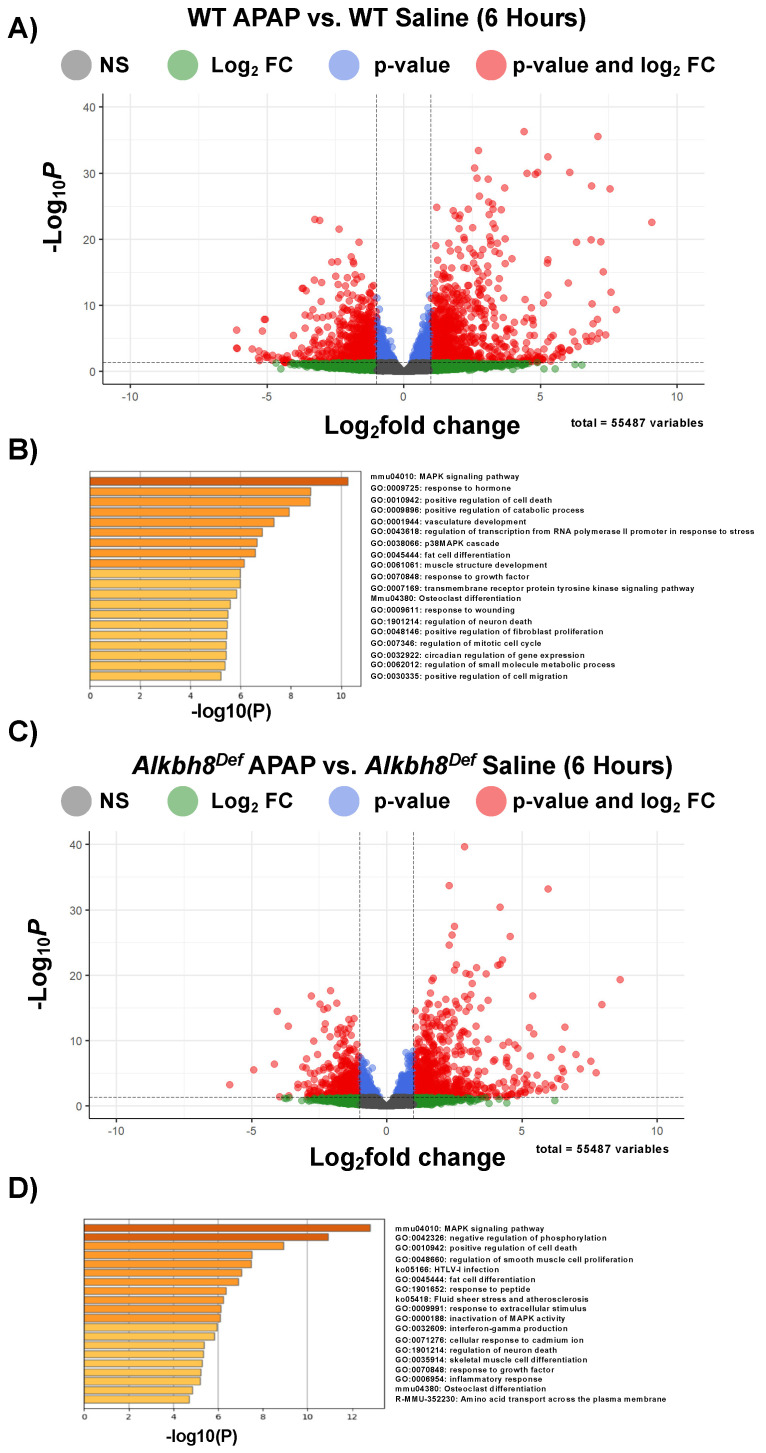
Next-generation mRNA sequencing analysis of the transcriptional response to APAP. WT and *Alkbh8^Def^* mice (N = 3) were left untreated or exposed to a single dose of APAP and livers were harvested after 6 h and RNA was purified and subject to mRNA-seq. Enhanced volcano plots from mRNA-seq data were generated for each of the comparisons and metascape analysis were performed for log_2_ FC > 2.0 and *p_adj._* values ≤ 0.05 for (**A**,**B**) WT APAP vs. WT Saline and (**C**,**D**) *Alkbh8^Def^* APAP vs. *Alkbh8^Def^* Saline.

**Figure 3:**
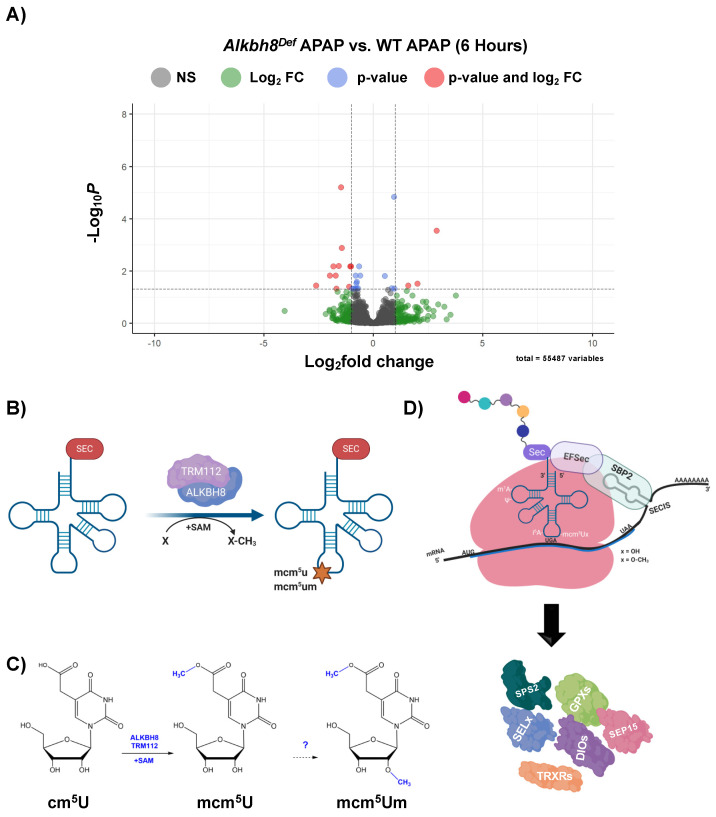
WT and *Alkbh8^Def^* livers have a similar transcriptional response to a single dose of APAP. mRNA-seq data for (**A**) *Alkbh8^Def^* APAP vs. WT APAP was compared using an enhanced volcano plot. (**B**) Post-transcriptional regulation of translation by Alkbh8 and epitranscriptomic marks. Alkbh8 requires the small accessory protein Trm112 and S-adenosylmethionine (SAM) to modify the wobble position of tRNA^Sec^ (**C**) from cm^5^U to mcm^5^U, which is then further modified to the ribose-methylated derivative mcm^5^Um. (**D**) Stop-codon recoding utilizes mcm^5^U and mcm^5^Um in tRNA^Sec^ to decode UGA to produce selenoproteins. In addition to Alkbh8-catlyzed epitranscriptomic marks, elongation factors, a selenocysteine insertion sequence (SECIS), accessory proteins, and an internal UGA stop codon are used to promote the translation of selenocysteine containing proteins.

We next analyzed gene expression using mRNA-seq of livers from saline and APAP-injected mice. Differential transcript expression was first visualized using enhanced volcano plots, with a *p_adj._* values ≤ 0.05, from studies using a single injection of APAP (6 h). WT APAP vs. WT saline (Figure 2A) and *Alkbh8^Def^* APAP vs. *Alkbh8^Def^* saline (Figure 2C) both show an extensive change in gene expression, with many transcripts significantly increased in response to APAP. These changes can be due to increased gene expression or decreased mRNA turnover. Enrichment of biological pathways in significantly up-regulated genes (log_2_fold change > 2.0 and *p_adj._* values ≤ 0.05) demonstrated that the following similar pathways were up-regulated in both WT (Figure 2B) and *Alkbh8^Def^* (Figure 2D) livers; MAPK signaling pathway, positive regulation of cell death, fat cell differentiation, osteoclast differentiation, response to growth factor, and regulation of neuron death. In general, both genotypes regulate groups of transcripts linked to stress responses. A comparison of mRNA seq results (Figure 3A) further supports the idea that the transcriptional response to APAP is very similar in WT and *Alkbh8^Def^* livers, where only 202 out of 55,487 measured transcripts were significantly different (*p* < 0.05) between the two genotypes. Transcriptional upregulated genes that had the largest significant difference in fold change include: odorant binding protein 2A (Obp2a), which aids in binding and transporting molecules with high affinity for aldehydes and large fatty acids [51], transmembrane protein 267 (Tmeme267), which present reported function involves protein binding [52], and a member of the MYC Proto-Oncogene (myc) family that is highly expressed in the brain (Bmyc) and functions as a transcriptional regulator. We also report similar gene transcript profiles when comparing WT and *Alkbh8^Def^* under saline control conditions (Suplemental Figure S1). The similarities in the transcriptional responses to APAP between the two genotypes was expected, as Alkbh8, along with its accessory protein Trm112, post transcriptionally regulate gene expression by modifying tRNA^Sec^ (Figure 3B–D) and other tRNAs. Alkbh8 can modify tRNA^Sec^, which is used in stop codon recoding, to drive the translation of 24 selenocysteine containing proteins in the Mus musculus genome (Figure 3D). Other regulatory components tied to stop codon recoding include the cis Sec insertion sequence (SECIS), a stem-loop structure located in coding regions and in 3′UTRs of eukaryotic genes [53].

### 3.3. The Epitranscriptome Is Extensively Regulated in Response to APAP and Disrupted in Alkbh8^Def^ Livers

**Figure 4:**
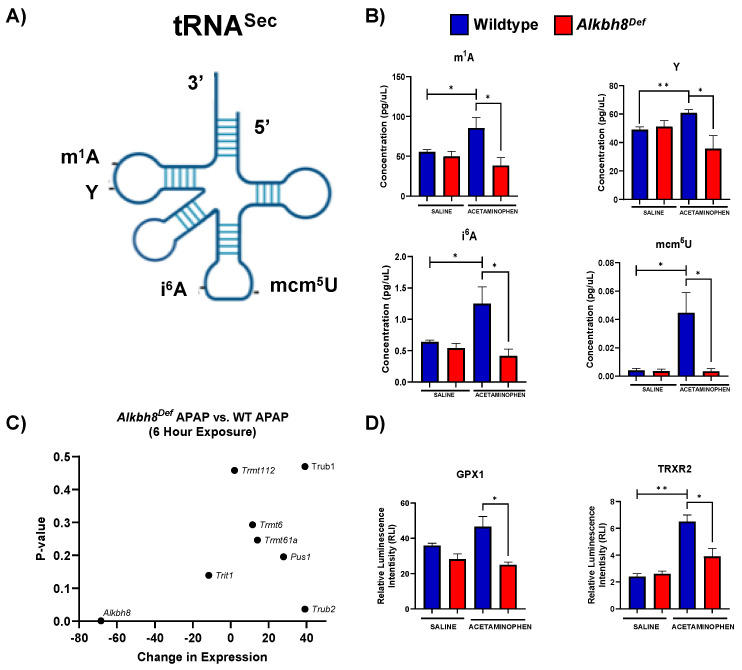
Multiple RNA modifications on tRNA for selenocysteine and other tRNAs are differentially regulated in WT and *Alkbh8^Def^* livers in response to a single dose of APAP. (**A**) Epitranscriptomic marks found on tRNA^Sec^. (**B**,**C**) WT and *Alkbh8^Def^* mice (N = 3) were exposed to a single dose of APAP and livers were harvested 6 h after dosing. (**B**) Modifications that occur throughout tRNA^Sec^ and other tRNAs were measured using LC-MS/MS. (**C**) Writer enzyme gene count changes in expression vs. p-value determined. (**D**) Selenoprotein levels (N = 3) were evaluated using the ProteinSimple WES system. Statistical significance was determined using an unpaired *t*-test with (* *p* < 0.05, ** *p* < 0.01).

We next analyzed for differences in post transcriptional regulation by quantifying epitranscriptomic marks in RNA from WT and *Alkbh8^Def^* livers. We used LC-MS/MS based approaches to analyze RNA modification levels in our purified small RNA samples. We note that the tRNA pool we analyzed has all isoacceptors and we did not analyze RNA modifications on specific tRNAs. It is interesting that tRNA^Sec^ possesses four different modifications (Figure 4A) that are catalyzed by either Alkbh8 (mcm^5^U), Trmt61A/Trm6 (m^1^A), Pus4 (Ψ), and Trit1 (i^6^A), and all four RNA modifications (mcm^5^U, m^1^A, Ψ, i^6^A) are increased in WT livers in response to APAP with these increases absent in *Alkbh8^Def^* livers (Figure 4B). The APAP induced increases in mcm^5^U, m^1^A, Ψ, and i^6^A could be due to increases throughout the pool of all tRNA isoacceptors, or could be due to increases on specific isoacceptors, with future studies needed to specifically determine. WT livers had significantly increased the concentration of the m^1^A modification from 55.51 pg/µL to 85.54 pg/µL, whereas the *Alkbh8^Def^* livers showed a decreased concentration from saline of 49.94 pg/µL to 38.48 pg/µL after APAP exposure. Similarly, Ψ was increased in WT saline (49.12 pg/µL) to WT APAP (60.76 pg/µL) conditions and showed a decreased concentration in *Alkbh8^Def^* saline (51.10 pg/µL) when compared to *Alkbh8^Def^* APAP (35.71 pg/µL) exposed liver tissue. The i^6^A modification was also dysregulated in the *Alkbh8^Def^* mice, where we measured a decreased concentration of 0.54 pg/µL in saline verse 0.42 pg/µL for APAP exposure. WT liver tissue had increased i^6^A modification from 0.64 pg/µL in saline conditions to 1.25 pg/µL after APAP exposure. Finally, the Alkbh8 dependent mcm^5^U modification was measured to have minimal change in concentration in the *Alkbh8^Def^* liver tissue between saline (0.0036 pg/µL) and APAP (0.0035 pg/µL) conditions, unlike the WT liver tissue where we measured a 10-fold increase from 0.004 pg/µL to 0.045 pg/µL in saline and APAP conditions, respectively. Using our mRNA-seq data we have observed that transcript levels for some writers are slightly dysregulated under APAP conditions in *Alkbh8^Def^* relative to WT (Figure 4C). The failure to increase epitranscriptomic marks in response to APAP in *Alkbh8^Def^* liver tissue predicts that selenoproteins levels would be affected. We quantified the protein levels Gpx1 and TrxR2 levels (Figure 4D), and, similar to Gpx3 (Figure 1D), Gpx1 and TrxR2 were increased in WT livers exposed to APAP; this stress induced increase in protein levels was absent in *Alkbh8^Def^* livers. Gpx4, TrxR1 and selenoprotein S (SelS) also failed to respond to APAP treatment in *Alkbh8^Def^* animals (Supplemental Figure S2).

**Figure 5:**
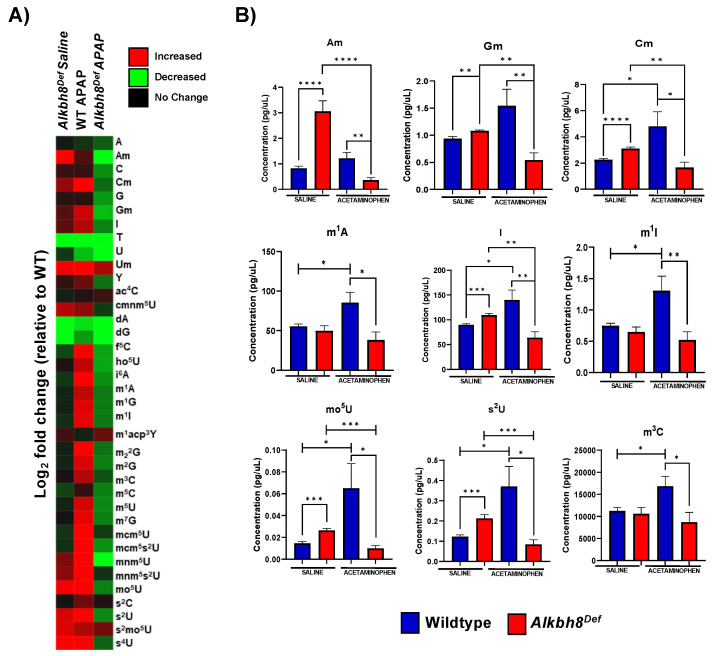
The epitranscriptome is differentially regulated in WT and *Alkbh8^Def^* livers in response to a single dose of APAP. (**A**) 37 epitranscriptome marks were measured with LC-MS/MS and the log_2_fold change was normalized relative to WT saline data and shown as a heat map. (**B**) Data for each modification under the four conditions was compared for statistical significance (N = 3) using an unpaired *t*-test with (* *p* < 0.05, ** *p* < 0.01, *** *p* < 0.001 and **** *p* < 0.0001).

We also measured 33 other tRNA modifications using LC-MS/MS and compared their levels, relative to WT saline, using a heat map (Figure 5A). The WT response to APAP resulted in the observation of 22 RNA modifications increased, whereas in the *Alkbh8^Def^* livers we have 13 modifications decreased (Table S1). In addition, the *Alkbh8^Def^* livers had 17 modifications decreased in saline control conditions, relative to WT saline (Supplemental Table S1A). Individual bar graphs of representative tRNA modifications from the heat map show mean values with standard error around the mean in WT and *Alkbh8^Def^* liver tissue under saline and APAP exposure conditions (Figure 5B). Other modifications are shown in (Supplemental Figure S3). The tRNA modifications that were the most downregulated in the *Alkbh8^Def^* APAP samples were; mnm^5^U (−6.4 log_2_fold change, *p* < 0.05) and mcm^5^U (−3.7 log_2_fold change, *p* < 0.05). Our epitranscriptomic data provides strong support for the idea that a single acute toxic dose of APAP promotes significant changes in many tRNA modifications on many different tRNA isoacceptors. Below we explore whether chronic APAP exposure promote changes to the epitranscriptome.

### 3.4. The Alkbh8 Writer Deficient Mice Respond to Chronic APAP with Altered Gene Expression and Have Increased Markers of Damage

**Figure 6:**
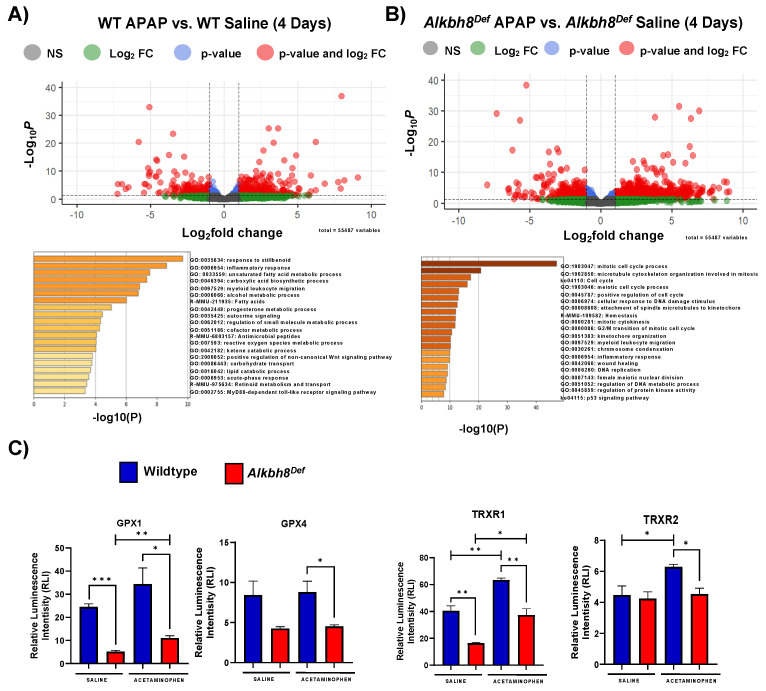
Writer deficient mice have altered gene expression and response due to altered epitranscriptomic response. WT (N = 6) and *Alkbh8^Def^* (N = 6) mice were IP injected daily with APAP. (**A**–**C**) Tissue was harvested at day 4 and RNA and protein were purified for analysis. (**A**,**B**) Enhanced volcano plots for mRNA-seq data were generated for each of the comparisons and metascape analysis was performed for log_2_ FC > 2.0 and *p_adj._* values ≤ 0.05. (**A**) WT APAP vs. WT Saline and (**B**) *Alkbh8^Def^* APAP vs. *Alkbh8^Def^* Saline. (**C**) Selenoprotein levels were evaluated using the ProteinSimple WES system. Statistical significance (N = 3) was determined using an unpaired *t*-test with (* *p* < 0.05, ** *p* < 0.01, and *** *p* < 0.001).

We analyzed mRNA from mice on the 4th day of daily APAP treatments (Supplemental Figures S4 and S5). mRNA sequencing experiments revealed many differences in gene regulation between WT and *Alkbh8^Def^* in response to APAP. The comparison of mRNA-seq data from WT APAP vs. WT saline identified significantly upregulated genes (*p* ≤ 0.05) in pathways related to response to stillbenoid, inflammatory response and unsaturated fatty acid metabolic process (Figure 6B). The *Alkbh8^Def^* APAP vs. *Alkbh8^Def^* Saline mRNA-seq comparison shows significantly (*p* ≤ 0.05) upregulated genes that are enriched for pathways related to mitotic cell cycle process, cellular response to DNA damage stimulus, and positive regulation of cell cycle (Figure 6C). A comparison of saline controls between WT and *Alkbh8^Def^* show very few differences in transcript levels at the 4-day time point (Supplemental Figure S4). However, unlike the single APAP dose, the daily doses of APAP promoted many differences in gene regulation between WT and the *Alkbh8^Def^* liver tissue (Supplemental Figure S5A). Pathways specific to cell division, regulation of cell cycle process and cellular response to DNA damage stimulus were significantly (*p* ≤ 0.05) enriched in *Alkbh8^Def^* APAP vs. WT APAP comparison (Supplemental Figure S5B), supporting the idea that there is more stress and DNA damage in *Alkbh8^Def^* livers compared to WT. The same selenoproteins that were measured for the 6 h timepoint were quantitated after 4 days of daily doses of APAP in liver tissue. Gpx1 expression in the WT mice show an increase in the APAP exposure group, and relative to WT the *Alkbh8^Def^* liver tissue shows decreased Gpx1. While Gpx4 was not increased in WT after APAP, there was a significant decrease in the expression of Gpx4 in the *Alkbh8^Def^* liver relative to WT. Both TrxR1 and TrxR2 protein levels were increased in WT APAP samples in response to saline, which was not observed in *Alkbh8^Def^* livers (Figure 6C, Supplemental Figure S6).

Results of LC-MS/MS analysis confirmed the disrupted response of many tRNA modifcaitons under chronic APAP condtions. In response to APAP the WT liver tissue showed significant increases in six modifications, and decreases in two modifications relative to WT saline. The *Alkbh8^Def^* mice had nine modifications significantly decreased after APAP exposure and five increased, relative to *Alkbh8^Def^* saline. The modification that had a similar response to acute conditions was m^1^A, with WT liver showing increases after APAP exposure and *Alkbh8^Def^* liver showing decreases. Many uridine modifications were measured to be significantly dysregulated in the *Alkbh8^Def^* liver tissue compared to WT after daily APAP exposure including: m^5^U, mcm^5^s^2^U, mnm^5^U, mnm^5^s^2^U, and s^2^U. It is interesting to note that s^2^U was measured to be increased after daily APAP exposure in *Alkbh8^Def^* mice compared to WT. Adenosine-based modifications m^1^A, and m^6^A were also measured to be dysregulated in the *Alkbh8^Def^* liver after daily APAP exposure. All measured modifications within the chronic timeline are shown in (Supplemental Figure S7) and (Supplemental Table S1B).

## 4. Discussion

Hepatoxic effects of APAP toxicity have been widely reported which include oxidative stress, impaired mitochondrial function, lipid peroxidation, nuclear DNA fragmentation and necrotic cell death [54,55,56,57,58]. ROS production from the bioactivation of APAP can promote DNA and protein damage [41,59,60,61]. The *Alkbh8^Def^* mice were unable to respond to the same stress load as their WT counterparts, and our work supports the idea that the epitranscriptomic writer, Alkbh8, has a protective role against APAP toxicity. The protection comes at the levels of tRNA modification and translation, with Alkbh8 catalyzing tRNA modifications and translationally regulating the production of stress response proteins. Wobble U writers in yeast, mice and humans have previously been shown to play key roles in responding to stress by enhancing the translation of ROS detoxification, DNA damage response and metabolic proteins [62,63,64,65]. For example, deficiencies in the mcm^5^U writer tRNA methyltransferase 9 (Trm9) in yeast sensitize cells to DNA damaging agents and disrupt cell cycle transitions, with decreased translation of ribonucleotide reductase genes driving these phenotypes [7,66]. Knockdown of human *ALKBH8* in HEK293 cells sensitize cells to ionizing radiation and disruption of mouse Alkbh8 sensitizes cells to the mitochondrial poison rotenone, due in part to the decrease in ROS detoxification enzymes [17,21]. Further it has been reported that there are increased markers of senescence in *Alkbh8^Def^* MEFs [38], highlighting a role for wobble U in regulating cell states linked to aging and disease. Notably, dysregulated expression of wobble U writers and their corresponding enzymes have also been linked to the etiology of cancer [4,67,68,69,70,71]. Wobble U writers play important translational regulatory roles that protect normal cells and organs from stress, and our studies highlight that the wobble U writer Alkbh8 helps regulate the response to APAP toxicity.

### 4.1. Translational Regulation Is a Key Response to APAP

Protein kinase cascades are well described responses to APAP, as it activates the c-Jun-N-terminal kinase (JNK) through the sequential protein phosphorylation of mitogen-activated protein kinases (MAPK) [72]. JNK is a critical member of the MAPK family and considered a control point between physiological and pathological status. The activation of JNK has been shown to be important in biological processes including stress reaction, cellular differentiation, and apoptosis [72,73]. The activated JNK can translocate to the mitochondria and binds to an outer membrane protein known as Sab, which is also phosphorylated. JNK binding to Sab leads to further ROS generation and sustains the activation of JNK leading to the induction of the mitochondrial permeability transition (MPT) resulting in the mediation of hepatocyte necrosis [74]. *c-Myc* is induced by APAP and encompasses one of the most significant protein networks that are associated with liver injury [75]. Beyer et al., presented gene ontology analysis of transcripts that significantly correlate with liver necrosis 6 h post-APAP exposure and the resulting biological pathways included programmed cell death, response to wounding, and inflammatory response [75]. These biological pathways are upregulated in both genotypes of mice after 6 hr APAP exposure in our study, when compared to their corresponding saline control. Our transcriptional studies support the idea that both WT and *Alkbh8^Def^* mice respond to APAP toxicity similarly at the mRNA level. The transcriptional data suggests that both genotypes have similar regulation of transcript production and turn-over.

Our study is unique in that we describe a change in RNA modification regulated protein expression in response to APAP stress. Members of the Gpx and TrxR selenoprotein families are known to be critical for signaling and aiding in protection and reduction of harmful oxidants [17,36,76]. Gpx and TrxR selenoproteins have also been shown to be key regulators of mitochondrial oxidative stress. Inhibition of these antioxidant enzymes with Auranofin in non-cancerous human Swan-71 cells resulted in reduction of cell viability, mitochondrial respiration, and increased oxidative stress. Selenium supplementation was able to restore Gpx and TrxR selenoprotein activity which subsequently decreased ROS production and increased cell viability [77]. These and other studies [78,79] confirm the important role of Gpx and TrxR selenoproteins as key response regulators to mitochondrial oxidant stress. Furthermore, APAP hepatoxicity has been reported to result in the formation of the potent oxidant peroxynitrite from superoxide and nitric oxide [80] which was subsequently observed to be predominately generated inside the mitochondria [81]. Therefore, the mitochondria are a significant source of oxidant stress in response to APAP. Our study demonstrated under non-stressed conditions the expression of selenoproteins are, relatively, similar between WT and *Alkbh8^Def^*. However, after APAP exposure we observe differences, with WT increasing expression of selenoproteins and *Alkbh8^Def^* having no change or decreased expression in measured selenoproteins. Our identification of a translational response to APAP, specific to selenoproteins that most likely respond to mitochondrial stress, highlights the importance of protein synthesis-based responses. Translational regulation is likely occurring in response to all pharmaceuticals that promote ROS stress, and studies in cancer models support the idea that translational regulation will be extended to many types of protein families and include regulation of the 20 standard amino acids [69].

### 4.2. RNA Modifications Are Systematically and Globally Re-Programmed in tRNAs in Response to Toxicants and Now Pharmaceuticals

tRNA modifications have been shown to be a critical part of epitranscriptomic reprogramming in response to stressors, chemicals, and physiological states in many organisms [7,17,62]. Our study highlights that APAP promotes a global reprogramming of RNA modifications. The tRNA modification, i^6^A was elevated in WT in response to APAP however this response was again disrupted in the *Alkbh8^Def^* mice. TRIT1 is responsible for modifying position 37 in the anticodon loop of tRNA^Sec^ and other tRNAs. The i^6^A modification is found on various cytoplasmic or mitochondrial tRNA. Cytoplasmic i^6^A modification occurs on tRNA^Ser^ and tRNA^Sec^, and mitochondrial tRNAs, mtRNA^Cys^, and mtRNA^Ser^ [82]. A reduced expression of *Trit1* can contribute to decreased selenoprotein expression [83], decreased translation of other cytoplasmic proteins and/or decreased mitochondrial translation, which could be occurring in the writer-deficient livers. The increase in mcm^5^U and other tRNA^Sec^ related modifications in WT livers in response to APAP are likely key drivers of selenoprotein synthesis.

After APAP exposure, WT liver responds by increasing m^1^A modification levels and this reprogramming is disrupted in the *Alkbh8^Def^* mice. m^1^A is present in cytosolic tRNA species at positions 9, 14, 22, 57, and 58, and, notably, on tRNA^Sec^ at position 58, as well as mitochondrial tRNA species at positions 9 and 58 [84]. The addition, the m^1^A modification is written by the catalytic protein Trmt61A and the RNA binding protein Trmt6 [16], while the eraser enzyme (Alkbh1) demethylates the m^1^A modification reverting it to its unmodified form [85]. The m^1^A modification aids in mitochondrial tRNA folding and ensures correct structure formation. The m^1^A58 tRNAs have a differential affinity for the elongation factor EF1A which delivers the tRNA into the A-site of the ribosome in protein synthesis [86]. Our data supports the idea that m^1^A plays an important translational role in protein synthesis in response to APAP and the deficiency of Alkbh8 dysregulates the epitranscriptomic reprogramming. Likely the m^1^A deficiency contributes to the decrease in selenoprotein synthesis, but its decrease could also be globally affecting protein synthesis in the cytoplasm and mitochondria. In addition, the m^1^A decrease could be promoting tRNA instability, which could account for the wide-spread decrease in RNA modifications in APAP treated *Alkbh8^Def^* livers.

The m^3^C modification was also significantly upregulated in WT livers after acute APAP exposure. The m^3^C modification is located at position 32 on various tRNA species as well as their corresponding isoacceptors including; tRNA^Arg^, tRNA^Met^, tRNA^Ser^, and tRNA^Thr^ and mt-tRNA^Met^, mt-tRNA^Ser^, and mt-tRNA^Thr^ [87]. The m^3^C modification is catalyzed by the methyltransferase-like protein 2 and 6 (METTL2 and METTL6) [88] and human METTL6 has been associated with regulating cellular growth, ribosomal occupancy, and pluripotency in hepatocellular carcinoma (HCC) [89]. Mettl6 knockout mice displayed altered metabolic activity via altered glucose homeostasis, changes in metabolic turnover, and decreased liver weight suggesting the impact *Mettl6* has on hepatic growth [89]. It is important to note that m^3^C Is not found on tRNA^Sec^ and its increase after APAP exposure suggests other proteins could be under translational regulation, in addition to selenoproteins.

### 4.3. Redox and Pharmaceuticals Responses Can Be Regulated by the Epitranscriptome

Oxidative stress is catalyzed by the disruption of redox homeostasis and the subsequent production of ROS. The modifications m^6^A, m^5^C and their respective modifying enzymes have been reported in various cellular models showing their protective role. Mettl3, the writer enzyme of m^6^A, has been shown to activate the Nrf2 antioxidant pathway in response to colistin-induced oxidative stress in mouse renal tubular epithelial cells [90]. Arsenite induced stress in human keratinocytes resulted in increased expression of other m^6^A writers, WTAP and Mettl14, which regulate changes in gene expression [91] as well as inhibiting p53 activation [91]. Eraser and reader enzymes of m^6^A also possess roles in oxidative stress response. FTO, the eraser enzyme, has implications in mitigating mitochondrial and lipogenesis-induced ROS in HEK293T and clear cell renal carcinoma cells [92]. FTO is a member of the Fe (II)- and α-KG-dependent dioxygenase enzyme family which requires electrons from Fe (II) and α-ketoglutarate to activate a dioxygene molecule [93]. The dioxygenases use oxygen as an electron acceptor to reduce methylation and to form formaldehyde and hydrogen peroxide which may suppress the overproduction of oxidative stress [94]. It is suggested that oxidative stress oxidizes Fe (II) to Fe (III) inhibiting the demethylase activity of eraser enzymes such as FTO [95,96]. However, overexpression of FTO in hepatocytes and myotubes has been linked to increasing lipogenesis and mitochondrial dysfunction leading to increased oxidative stress [97,98]. Reader enzymes YTHDF1-3 studies have also been shown to have various roles in recognizing oxidative stress signaling and promote regulation of antioxidant pathways, in addition to influencing mRNA stability and translation [99,100]. m^5^C writer enzymes have been shown in HeLa and colon cancer cell lines to upregulate NRF2 and induce cellular senescence upon oxidative stress exposure [101,102]. Together with our current Alkbh8 study, past studies highlight the importance of epitranscriptomic marks in response to stress and link mcm^5^U, m^1^A, m^3^C, m^6^A, and m^5^C as key signals regulating the response to ROS stress. Together they highlight there is likely a systems-based reprogramming of the epitranscriptome in response to stress. The observation that an extensive number of RNA modifications are dysregulated in the liver of *Alkbh8^Def^* mice is likely due to the increased ROS stress or mitochondrial dysfunction [17,36,38]. Studies in yeast have shown that the tRNA epitranscriptome is extensively re-programmed in response to H_2_O_2_ and other ROS-inducing agents [6,7,8]. Similarly, Alkbh8 deficiency has been linked to changes in mitochondrial physiology [38], which could also be driving the extensive epitranscriptomic reprograming.

## 5. Conclusions

**Figure 7:**
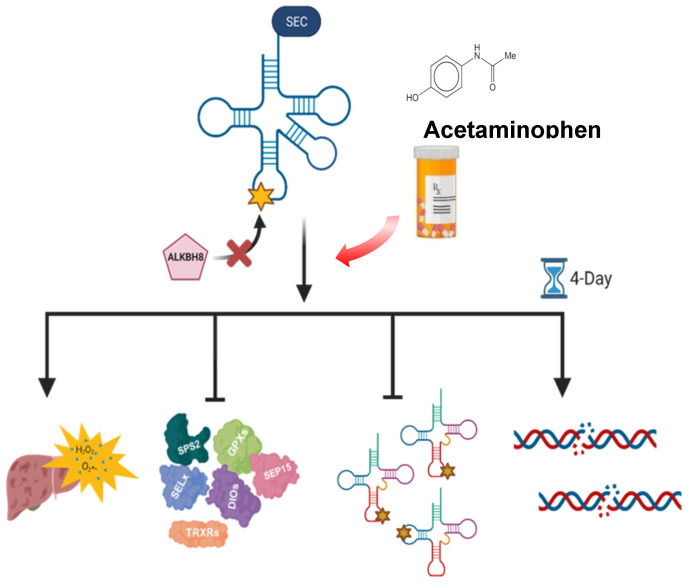
Model of APAP sensitivity in writer and modification dysregulated Alkbh8^Def^ livers. Alkbh8 plays a protective role against oxidative stress-induced APAP toxicity. *Alkbh8^Def^* mice experienced higher instances of oxidative stress, liver damage and overall decreased survival. The deficiency of *Alkbh8* results in the dysregulation of the epitranscriptome and decreased selenoprotein expression.

Our study highlights that APAP promotes global reprogramming of the epitranscriptome and its effects can be in part modulated by a single epitranscriptome writer (Figure 7). The deficiency of *Alkbh8* resulted in increased liver damage and oxidative stress in *Alkbh8^Def^* mice upon APAP exposure, which can be linked to a global disruption of epitranscriptomic reprogramming, a specific decrease in wobble U modification and the decreased translation of stress response proteins. A major finding of our study is that pharmaceutical stress promotes changes to the epitranscriptome and RNA modification enzymes can modulate outcomes. The efficacy of chemotherapeutics and immunotherapies have also been shown to be modulated by epitranscriptomic systems. METTL3 expression increases resistance to the chemotherapeutic agents 5-fluorouracil, gemcitabine, and cisplatin in pancreatic cancer, through activation of the MAPK signaling pathway [103]. The eraser enzyme, FTO, promotes melanoma tumorigenesis and resistance to immunotherapy, while decreased FTO expression increased IFN*y*-induced tumor cell killing and promoted beneficial PD-1 immunotherapy [104]. Similarly, decreased expression of the eraser ALKBH5 increased the efficiency of PD-1 therapy in both melanoma and colorectal cancer models by decreasing immunosuppressive cells [105]. Additionally, wobble U34 modifications catalyzed by ELP3/CTU1/2 have been shown to drive melanomas that express mutated BRAF, with subsequent resistance to anti-BRAF therapy caused by U34-dependent codon-biased translation of metabolic proteins [70]. It is important to note that many of the previously published studies showing that the efficacy of therapeutics can be modulated by epitranscriptomic marks were performed using in situ models and focus on agents specific to cancer treatment. Our study presents an in vivo model of how a writer can modulate pharmaceutical stress resulting from a pain reliever and suggests that epitranscriptomic modulation could augment other therapeutics. Overexpression of *Alkbh8* or increased wobble modifications in tRNA^Sec^ could limit APAP toxicity and provide an increased protective role against oxidative stress. Notably, selenium supplementation has been shown to promote increased wobble modification of tRNA^Sec^ and promote selenoprotein expression [106], and it could offer an effective epitranscriptomic enhancement strategy to counteract APAP overdose.

## Data Availability

Raw data files for protein studies are found in supplementary materials. We have deposited mRNA-seq data in the Gene Expression Omnibus, GSE169155, at: https://www.ncbi.nlm.nih.gov/geo/query/acc.cgi?acc=GSE169155 (deposited on 9 January 2021).

## Supplementary Materials

The following supporting information can be downloaded at: https://www.mdpi.com/article/10.3390/genes13030421/s1, Table S1: Measured epitranscriptomic marks in mouse liver tissue after APAP; Table S2: WES raw data for all proteins analyzed in 6 h APAP exposure experiment; Table S3: WES raw data for all proteins analyzed in 4 day APAP exposure experiment; Table S4: All measured epitranscriptomic marks after daily 600 mg/kg APAP for 4 days; Figure S1: Transcripts regulated in *Alkbh8^Def^* Saline vs. WT Saline mice in the 6 h APAP experiment; Figure S2: Selenoproteins measured after 6 h APAP exposure; Figure S3: Remainder of measured epitranscriptomic marks in mouse liver tissue after APAP (6 h); Figure S4: Transcripts regulated in *Alkbh8^Def^* Saline verse WT Saline mice in the 4 day APAP experiment; Figure S5: Transcripts regulated in *Alkbh8^Def^* APAP verse WT APAP in the 4 day experiment; Figure S6: Selenoprotein S expression elevated in *Alkbh8^Def^* liver tissue after 4 day APAP exposure; Figure S7: tRNA modifications measured after 4 day APAP exposure.
