# Supplementary material for: Epitranscriptomic Reprogramming Is Required to Prevent Stress and Damage from Acetaminophen
Source: Genes (Basel). 2022 Feb 25;13(3):421. doi: 10.3390/genes13030421 (PMC8955276; doi:10.3390/genes13030421)

Supplemental Tables

Supplemental Table S1A. Measured epitranscriptomic marks in mouse liver tissue after APAP (6 hours). Epitranscriptomic mark changes and comparisons between WT and Alkbh8^{Def} liver tissue post 6-hour exposure to 600 mg/kg of APAP comparisons between WT and Alkbh8^{Def} liver tissue post 6-hour exposure to 600 mg/kg of APAP with reported statistical significance of biological replicates (N = 3) measured by an unpaired t-test.

tRNA Modification	*Alkbh8^{Def}* Saline vs. WT Saline, Log_2 Fold Change	WT APAP vs. WT Saline, Log_2 Fold Change	*Alkbh8^{Def}* APAP vs. WT Saline, Log_2 Fold Change	*Alkbh8^{Def}* APAP vs. WT APAP, Log_2 Fold Change	*Alkbh8^{Def}* APAP vs. *Alkbh8^{Def}* Saline, Log_2 Fold Change	WT APAP vs. WT Saline p-Value	*Alkbh8^{Def}* Saline vs WT Saline p-Value	*Alkbh8^{Def}* APAP vs. WT APAP p-Value	*Alkbh8^{Def}* APAP vs. *Alkbh8^{Def}* Saline p-Value
A	0.0	-0.1	-0.3	-0.2	-0.28	0.0340	0.0470	0.0460	0.0037
Am	1.6	0.2	-1.5	-1.7	-3.08	0.0700	0.0001	0.0022	0.000004
C	0.2	0.1	-0.5	-0.7	-0.67	0.0920	0.0005	0.0180	0.0160
Cm	0.6	1.2	-0.4	-1.5	-0.91	0.3010	0.0001	0.0100	0.0020
G	0.1	0.1	-0.6	-0.6	-0.63	0.0680	0.0011	0.0084	0.0088
Gm	0.3	0.8	-0.7	-1.5	-1.00	0.3210	0.0032	0.0042	0.0006
I	0.3	0.7	-0.5	-1.1	-0.77	0.2840	0.0001	0.0024	0.0011
T	-6.7	-1.2	-7.7	-6.5	-1.02	0.0000	0.0003	0.0440	0.1250
U	-0.1	-0.7	-3.8	-3.1	-3.65	0.3060	0.2180	0.0034	0.00001
Um	0.9	1.4	0.6	-0.8	-0.30	0.3440	0.1110	0.3100	0.3350
Y	0.1	0.4	-0.4	-0.8	-0.52	0.0046	0.2080	0.0090	0.0740
ac^4C	-0.1	0.1	0.2	0.1	0.22	0.0750	0.4190	0.0036	0.1100
$cmnm^5U$	0.7	0.5	-0.2	-0.7	-0.86	0.1700	0.0240	0.0620	0.0110
dA	-4.6	-0.9	-5.5	-4.6	-0.90	2.10E-13	0.0000004	0.0210	0.1240
dG	-4.8	-0.6	-5.5	-5.0	-0.76	0.0000001	0.00002	0.0240	0.1340
f^5C	-0.2	1.0	-0.6	-1.6	-0.40	0.0190	0.3690	0.0190	0.3130
ho^5U	0.0	0.7	-0.6	-1.4	-0.67	0.0110	0.4720	0.0230	0.1750
i^6A	-0.2	1.1	-0.5	-1.6	-0.36	0.0001	0.2530	0.0055	0.1870
m^1A	-0.1	0.7	-0.4	-1.2	-0.38	0.0001	0.4010	0.0053	0.1710
m^1G	-0.1	0.9	-0.4	-1.3	-0.38	0.0001	0.4000	0.0060	0.1730
m^1I	-0.1	0.9	-0.4	-1.3	-0.31	0.0001	0.3120	0.0045	0.2140
$m^1acp^3\psi U$	0.2	-0.1	0.4	0.4	0.17	0.0350	0.2690	0.0490	0.1690
$m^{22}G$	-0.1	1.2	-0.4	-1.6	-0.34	0.0036	0.4110	0.0095	0.1890
m^2G	-0.1	0.8	-0.5	-1.3	-0.39	0.0001	0.3830	0.0058	0.1660
m^3C	0.0	0.7	-0.3	-0.9	-0.28	0.0001	0.4760	0.0100	0.2390
m^5C	-0.2	0.1	-0.5	-0.6	-0.26	0.0002	0.1770	0.0340	0.2620
m^5U	-0.1	0.8	-0.5	-1.3	-0.44	0.0001	0.3940	0.0042	0.1340
m^7G	0.0	0.9	-0.5	-1.4	-0.46	0.0003	0.4990	0.0046	0.1220
mcm^5U	-0.1	3.5	-0.2	-3.7	-0.04	0.0370	0.4310	0.0056	0.4820
mcm^5s^2U	-0.1	1.0	-0.5	-1.6	-0.41	0.0001	0.2990	0.0066	0.1500
mnm^5U	0.4	4.7	-1.7	-6.4	-2.14	0.0390	0.0150	0.0290	0.000001

**mnm^{5}s^{2}U**	0.5	3.1	-0.1	-3.2	-0.62	0.3430	0.0440	0.0290	0.0500
**mo^{5}U**	0.9	2.3	-0.5	-2.7	-1.41	0.2330	0.00004	0.0130	0.0001
**s^{2}C**	0.0	0.4	0.0	-0.3	0.04	0.1170	0.4970	0.1220	0.4510
**s^{2}U**	0.8	1.6	-0.5	-2.1	-1.33	0.4040	0.0001	0.0067	0.0002
**s^{2}mo^{5}U**	0.9	0.6	0.4	-0.2	-0.44	0.3310	0.0260	0.2360	0.1030
**s^{4}U**	1.4	1.8	-0.3	-2.2	-1.74	0.0007	0.00002	0.0180	0.0001

Supplemental Table S1B. Measured tRNA modifications in mouse liver tissue after daily dose of APAP (4 Days). Calculations for each epitranscriptomic mark and comparisons between WT and Alkbh8^{Def} liver tissue post daily 4 Day exposure to 600 mg/kg of APAP.

tRNA Modifica-tion	*Alkbh8^{Def}* Saline vs. WT Saline, Log_2 Fold Change	WT APAP vs. WT Saline, Log_2 Fold Change	*Alkbh8^{Def}* APAP vs. WT Saline Log_2 Fold Change	*Alkbh8^{Def}* APAP vs. WT APAP Log_2 Fold Change	*Alkbh8^{Def}* APAP vs. *Alkbh8^{Def}* Saline Log_2 Fold Change	WT APAP vs. WT Saline, p-Value	*Alkbh8^{Def}* *Saline* vs WT Saline, p-Value	*Alkbh8^{Def}* APAP vs. WT APAP, p-Value	*Alkbh8^{Def}* APAP vs. *Alkbh8^{Def}* Saline, p-Value
A	3.2E-03	-0.23	-0.32	-8.7E-02	-0.32	3.8E-04	0.46	0.11	1.7E-06
Am	-0.90	0.63	-0.41	-1.04	0.49	0.34	0.27	0.27	0.38
C	-0.10	0.10	-4.8E-02	-0.15	5.5E-02	0.20	0.17	0.05	0.18
G	-3.6E-02	2.2E-02	-8.4E-02	-0.11	-4.8E-02	0.33	0.23	5.4E-03	7.3E-02
I	-0.12	0.19	1.6E-02	-0.17	0.14	0.20	0.28	0.13	0.12
T	-6.0E-03	-1.18	0.18	1.35	0.18	9.5E-02	0.50	3.8E-02	0.35
U	0.20	1.26	1.50	0.24	1.30	2.3E-04	0.21	0.13	5.3E-06
Um	-1.47	-4.21	-4.12	8.7E-02	-2.66	9.9E-05	8.4E-03	0.36	2.0E-02
Y	-0.12	0.12	-0.13	-0.25	-6.9E-03	0.27	0.27	2.5E-02	0.46
dA	0.24	1.04	0.60	-0.44	0.35	1.3E-07	0.23	6.6E-03	0.14
dG	0.40	0.82	0.71	-0.11	0.31	2.0E-06	4.7E-02	0.22	7.3E-02
f^5C	-0.25	0.28	-0.08	-0.36	0.17	0.18	0.21	6.0E-02	0.18
ho^5U	-0.42	-0.95	-0.14	0.80	0.28	6.1E-02	0.17	0.10	0.26
i^6A	0.15	0.03	-0.10	-0.13	-0.25	0.42	0.12	1.5E-02	8.5E-06
m^1A	0.22	0.25	-0.04	-0.29	-0.26	1.0E-02	1.5E-02	6.7E-05	3.0E-05
m^1G	-5.9E-02	7.9E-02	2.7E-02	-5.2E-02	8.6E-02	0.26	0.28	0.31	0.14
m^1I	-5.42	-5.52	-5.64	-0.12	-0.21	0.17	0.17	0.43	0.38
$m^1acp^3\Psi U$	-1.41	0.66	-0.22	-0.87	1.19	7.9E-02	6.4E-02	2.3E-02	8.2E-02
m^22G	0.92	1.50	1.51	1.4E-02	0.59	1.1E-12	7.1E-03	0.28	3.6E-03
m^2G	1.90	1.58	1.14	-0.44	-0.76	3.8E-02	3.7E-02	0.28	0.18
m^3C	0.11	0.12	-8.1E-02	-0.20	-0.19	0.15	0.15	4.8E-04	6.6E-05
m^5C	0.08	0.09	-0.07	-0.15	-0.15	0.23	0.23	1.6E-02	4.7E-03
m^5U	8.9E-02	-4.3E-02	-0.19	-0.15	-0.28	0.29	0.10	3.6E-03	9.8E-07
m^6A	-0.96	0.24	-0.36	-0.59	0.60	0.31	5.4E-02	6.5E-02	4.7E-02
mcm^5U	-1.36	-1.08	-0.94	0.13	0.41	0.10	7.5E-02	0.41	0.26
mcm^5s^2U	0.12	0.16	-0.05	-0.21	-0.17	9.4E-02	0.16	1.0E-03	6.0E-03
mnm^5U	-0.11	0.28	-8.1E-02	-0.36	3.4E-02	0.13	0.29	4.1E-02	0.38
mnm^5s^2U	9.1E-02	7.2E-04	-0.37	-0.37	-0.46	0.50	0.20	1.1E-03	3.6E-05
mo^5U	-0.36	0.49	0.25	-0.24	0.61	0.17	0.27	0.20	8.1E-03
s^2C	0.08	-0.08	5.6E-02	0.14	-2.9E-02	0.17	0.20	3.9E-02	0.38
s^2U	-2.32	-1.11	0.99	2.10	3.30	5.3E-02	1.5E-03	9.3E-03	2.2E-03
s^2mo^5U	-0.62	-0.61	1.2E-03	0.61	0.63	0.16	0.16	0.17	0.17
s^4U	-0.24	0.29	6.1E-03	-0.28	0.24	0.14	0.19	7.6E-02	5.6E-02

Supplemental Table S2. WES raw data for all proteins analyzed in 6 hour APAP exposure experiment. Protein quantitation data was normalized to housekeeping protein, GAPDH, and normalized corrected area analysis setting was set to 100 on ProteinSimple Compass Software.

Sample	Primary Antibody	Capillary	Peak	Name	Position	MW (kDa)	Height	Area	% Area	Corr. Area	Width	S/N	Baseline
WT521Saline	GPX1/GAPH	C1:3	1	GPX1	449	28	50596.4	540734	27.9	38.6	10	1017.5	607.3
WT521Saline	GPX1/GAPH	C1:3	2	GAPDH	494	39	157614.7	1400424	72.1	100	8.3	3291.7	707.3
WT522Saline	GPX1/GAPH	C1:5	1	GPX1	443	28	77534.2	673172	25.8	34.8	8.2	1331.1	900.1
WT522Saline	GPX1/GAPH	C1:5	2	GAPDH	489	39	249773.5	1933917	74.2	100	7.3	4359.3	1097.7
WT526Saline	GPX1/GAPH	C1:6	1	GPX1	442	28	110140.8	930117	25.5	34.2	7.9	2434.5	649.1
WT526Saline	GPX1/GAPH	C1:6	2	GAPDH	487	38	354684.5	2718060	74.5	100	7.2	7913.4	727.1
Alkbh8^{Def}*549*-/- Saline	GPX1/GAPH	C1:4	1	GPX1	443	28	50636.3	483065	18.1	22	9	1011.6	553.6
Alkbh8^{Def}*549*-/- Saline	GPX1/GAPH	C1:4	2	GAPDH	489	39	266771.8	2190817	81.9	100	7.7	5488.5	633.9
Alkbh8^{Def}*550*-/- Saline	GPX1/GAPH	C1:9	1	GPX1	442	28	83423.8	696101	23.9	31.4	7.8	1984.4	603.8
Alkbh8^{Def}*550*-/- Saline	GPX1/GAPH	C1:8	3	GAPDH	488	39	266838.5	1939870	76.3	100	6.8	7070.2	719.5
Alkbh8^{Def}*551*-/- Saline	GPX1/GAPH	C1:8	2	GPX1	442	28	70059.8	601533	23.7	31	8.1	1866.6	655.8
Alkbh8^{Def}*551*-/- Saline	GPX1/GAPH	C1:9	2	GAPDH	488	39	307847.5	2214587	76.1	100	6.8	7342.2	666.9
WT525 APAP	GPX1/GAPH	C1:7	1	GPX1	444	28	123456.2	1025526	29.6	42	7.8	2493.1	732.5
WT525 APAP	GPX1/GAPH	C1:7	2	GAPDH	489	39	308642.7	2443044	70.4	100	7.4	6243.6	850.5
WT527 APAP	GPX1/GAPH	C1:10	1	GPX1	448	28	31985.4	320962	36.8	58.1	9.4	804.7	566.5
WT527 APAP	GPX1/GAPH	C1:10	2	GAPDH	495	38	74816.9	552157	63.2	100	6.9	1911.3	655.6
WT528 APAP	GPX1/GAPH	C1:11	1	GPX1	446	28	114804.1	1024475	28.3	39.4	8.4	2856.7	738.4
WT528 APAP	GPX1/GAPH	C1:11	2	GAPDH	491	38	317967.8	2601984	71.7	100	7.7	8071.3	909
Alkbh8^{Def}546-/- APAP	GPX1/GAPH	C1:2	1	GPX1	446	28	62161.4	535190	20.2	25.3	8.1	1418.1	588.8
Alkbh8^{Def}546-/- APAP	GPX1/GAPH	C1:2	2	GAPDH	492	39	276573.9	2117178	79.8	100	7.2	6386.6	698
Alkbh8^{Def}547-/- APAP	GPX1/GAPH	C1:4	1	GPX1	443	28	50636.3	483065	18.1	22	9	1011.6	553.6
Alkbh8^{Def}547-/- APAP	GPX1/GAPH	C1:4	2	GAPDH	489	39	266771.8	2190817	81.9	100	7.7	5488.5	633.9
Alkbh8^{Def}548-/- APAP	GPX1/GAPH	C1:13	1	GPX1	442	28	129755.5	1004248	21.6	27.5	7.3	2758.6	713.9
Alkbh8^{Def}548-/- APAP	GPX1/GAPH	C1:13	2	GAPDH	487	38	494639.9	3647564	78.4	100	6.9	10889.2	795.1
WT521Saline	GPX3/GAPDH	C1:14	1	GPX3	447	29	871.7	7367	0.2	0.2	7.9	17.1	497.4
WT521Saline	GPX3/GAPDH	C1:14	2	GAPDH	493	39	615300.4	4344585	99.8	100	6.6	15282.2	566.1
WT522Saline	GPX3/GAPDH	C1:15	1	GPX3	451	29	750.6	8679	0.7	0.7	10.9	15.8	581.9
WT522Saline	GPX3/GAPDH	C1:15	2	GAPDH	496	39	158649.9	1256936	99.3	100	7.4	3738	683.4
WT526Saline	GPX3/GAPDH	C1:16	1	GPX3	448	28	736.5	7740	0.4	0.4	9.9	9.8	804.4
WT526Saline	GPX3/GAPDH	C1:16	2	GAPDH	492	39	260322	2027896	99.6	100	7.3	4312.3	945
Alkbh8^{Def}*549*-/- Saline	GPX3/GAPDH	C1:17	1	GPX3	447	28	1032.6	9308	0.5	0.5	8.5	20.7	686.2
Alkbh8^{Def}*549*-/- Saline	GPX3/GAPDH	C1:17	2	GAPDH	492	39	245403.4	1823891	99.5	100	7	5113.5	816.7
Alkbh8^{Def}*550*-/- Saline	GPX3/GAPDH	C1:18	1	GPX3	447	28	2341.4	18086	0.6	0.6	7.3	40	537
Alkbh8^{Def}*550*-/- Saline	GPX3/GAPDH	C1:18	2	GAPDH	491	39	397971.1	3021578	99.4	100	7.1	8563.2	610.9
Alkbh8^{Def}*551*-/- Saline	GPX3/GAPDH	C1:19	1	GPX3	452	29	3279.2	37717	1.5	1.5	10.8	102.6	279
Alkbh8^{Def}*551*-/- Saline	GPX3/GAPDH	C1:19	2	GAPDH	494	39	311474.5	2482104	98.5	100	7.5	10771.4	319.9
WT525 APAP	GPX3/GAPDH	C1:6	1	GPX3	419	29	3139.2	41945	6.8	7.3	12.6	102.1	103.7
WT525 APAP	GPX3/GAPDH	C1:6	2	GAPDH	466	39	77871.3	571499	100	100	6.9	2617.6	118.3
WT527 APAP	GPX3/GAPDH	C1:22	1	GPX3	444	29	697.1	10269	3	3.1	13.8	7.6	864.2

WT527 APAP	GPX3/GAPDH	C1:22	2	GAPDH	493	38	48980.9	335193	97	100	6.4	876.5	974.2
WT528 APAP	GPX3/GAPDH	C1:7	1	GPX3	431	29	53774.6	437505	6.3	6.8	7.6	1818.5	390.2
WT528 APAP	GPX3/GAPDH	C1:7	2	GAPDH	486	39	951763.4	6480246	93.7	100	6.4	32570.6	427.5
Alkbh8^{Def}546-/- APAP	GPX3/GAPDH	C1:23	1	GPX3	426	25	51.8	316	0	0	5.7	1.2	703.5
Alkbh8^{Def}546-/- APAP	GPX3/GAPDH	C1:23	2	GAPDH	491	40	272274.9	1791455	100	100	6.2	3617.3	899.3
Alkbh8^{Def}547-/- APAP	GPX3/GAPDH	C1:24	1	GPX3	441	28	1018.1	8948	0.4	0.4	8.3	18.2	724
Alkbh8^{Def}547-/- APAP	GPX3/GAPDH	C1:24	2	GAPDH	491	40	332330.9	2243327	99.6	100	6.3	6317.6	859.9
Alkbh8^{Def}548-/- APAP	GPX3/GAPDH	C1:25	1	GPX3	447	29	2877.7	23376	1	1	7.6	57.9	812.9
Alkbh8^{Def}548-/- APAP	GPX3/GAPDH	C1:25	2	GAPDH	493	39	344421.8	2297862	99	100	6.3	6869.6	972.6
WT521Saline	GPX4/GAPDH	C1:2	1	GPX4	429	27	33883.8	292658	4.6	4.8	8.1	965.2	374
WT521Saline	GPX4/GAPDH	C1:2	2	GAPDH	485	39	868101.8	6061692	95.4	100	6.6	24993.2	510
WT522Saline	GPX4/GAPDH	C1:3	1	GPX4	431	27	18072.2	166662	5.2	5.5	8.7	590.6	364.6
WT522Saline	GPX4/GAPDH	C1:3	2	GAPDH	487	38	465954.5	3055311	94.8	100	6.2	14776	405
WT526Saline	GPX4/GAPDH	C1:4	1	GPX4	430	27	25443.4	262771	4	4.2	9.7	614.5	387.5
WT526Saline	GPX4/GAPDH	C1:4	2	GAPDH	488	40	836773	6229058	96	100	7	20888.3	458.4
Alkbh8^{Def}*549*-/- Saline	GPX4/GAPDH	C1:7	1	GPX4	431	27	53774.6	437505	6.3	6.8	7.6	1818.5	390.2
Alkbh8^{Def}*549*-/- Saline	GPX4/GAPDH	C1:7	2	GAPDH	486	39	951763.4	6480246	93.7	100	6.4	32570.6	427.5
Alkbh8^{Def}*550*-/- Saline	GPX4/GAPDH	C1:5	1	GPX4	429	27	29111.1	246364	5	5.3	8	906.6	338.9
Alkbh8^{Def}*550*-/- Saline	GPX4/GAPDH	C1:5	2	GAPDH	485	40	716638	4677900	95	100	6.1	21789.4	403.6
Alkbh8^{Def}*551*-/- Saline	GPX4/GAPDH	C1:6	1	GPX4	429	27	35695.8	300060	5	5.2	7.9	1537.5	388.6
Alkbh8^{Def}*551*-/- Saline	GPX4/GAPDH	C1:6	2	GAPDH	486	39	853578.7	5741046	95	100	6.3	36274.7	464.3
WT525 APAP	GPX4/GAPDH	C1:11	1	GPX4	419	28	18458.3	215257	7.4	8	11	419	497
WT525 APAP	GPX4/GAPDH	C1:11	2	GAPDH	470	37	368568.3	2701793	98.5	100	6.9	8941.1	553
WT527 APAP	GPX4/GAPDH	C1:8	1	GPX4	430	27	22958.7	203449	4.1	4.3	8.3	1071.5	404.9
WT527 APAP	GPX4/GAPDH	C1:8	2	GAPDH	486	40	723083.5	4737719	95.9	100	6.2	33566.9	474.3
WT528 APAP	GPX4/GAPDH	C1:10	1	GPX4	415	28	18810.1	237075	3.6	3.7	11.8	439	613.7
WT528 APAP	GPX4/GAPDH	C1:10	2	GAPDH	466	39	874537.4	6363359	97.6	100	6.8	23392.2	758.7
Alkbh8^{Def}546-/- APAP	GPX4/GAPDH	C1:11	1	GPX4	432	27	31485.6	279504	4.7	4.9	8.3	1148.2	336.8
Alkbh8^{Def}546-/- APAP	GPX4/GAPDH	C1:11	2	GAPDH	488	40	852666.6	5659861	95.3	100	6.2	31036.4	368
Alkbh8^{Def}547-/- APAP	GPX4/GAPDH	C1:7	1	GPX4	431	27	53774.6	437505	6.3	6.8	7.6	1818.5	390.2
Alkbh8^{Def}547-/- APAP	GPX4/GAPDH	C1:7	2	GAPDH	486	39	951763.4	6480246	93.7	100	6.4	32570.6	427.5
Alkbh8^{Def}548-/- APAP	GPX4/GAPDH	C1:2	1	GPX4	429	27	33883.8	292658	4.6	4.8	8.1	965.2	374
Alkbh8^{Def}548-/- APAP	GPX4/GAPDH	C1:2	2	GAPDH	485	39	868101.8	6061692	95.4	100	6.6	24993.2	510
WT521Saline	TRXR1/GAPDH	C1:2	3	TRXR1	535	56	148489.3	1486319	16.5	19.8	9.4	2398.2	1361.4
WT521Saline	TRXR1/GAPDH	C1:2	1	GAPDH	489	40	1027393	7513838	83.5	100	6.9	17210.3	1337.6
WT522Saline	TRXR1/GAPDH	C1:3	2	TRXR1	535	55	74666.9	815199	14.6	17.1	10.3	970.4	1711
WT522Saline	TRXR1/GAPDH	C1:3	1	GAPDH	489	40	649420.2	4766565	85.4	100	6.9	8790.1	1602.2

WT526Saline	TRXR1/GAPDH	C1:4	2	TRXR1	534	56	114324	1168248	16.5	19.8	9.6	1477.9	1379.5
WT526Saline	TRXR1/GAPDH	C1:4	1	GAPDH	488	40	810967.8	5904967	83.5	100	6.8	10743.4	1362
Alkbh8^{Def}549-/- Saline	TRXR1/GAPDH	C1:5	2	TRXR1	534	55	99249.2	1118277	14.9	17.5	10.6	916.2	1599.3
Alkbh8^{Def}549-/- Saline	TRXR1/GAPDH	C1:5	1	GAPDH	488	39	852572	6385236	85.1	100	7	8359.5	1540.7
Alkbh8^{Def}550-/- Saline	TRXR1/GAPDH	C1:6	2	TRXR1	534	55	118215.5	1324038	14.5	16.9	10.5	1797.2	1762.6
Alkbh8^{Def}550-/- Saline	TRXR1/GAPDH	C1:6	1	GAPDH	487	39	1065369	7837010	85.5	100	6.9	17383.8	1631.5
Alkbh8^{Def}551-/- Saline	TRXR1/GAPDH	C1:8	2	TRXR1	534	55	121081.6	1312601	15.1	17.8	10.2	1329.1	1425.5
Alkbh8^{Def}551-/- Saline	TRXR1/GAPDH	C1:9	2	GAPDH	488	39	948778.2	6728725	83.9	100	6.7	15428.6	1386.7
WT525 APAP	TRXR1/GAPDH	C1:14	4	TRXR1	514	55	105175.6	1248680	17.6	21.4	11.2	2167.8	1555.3
WT525 APAP	TRXR1/GAPDH	C1:14	2	GAPDH	467	39	835816.1	5831180	100	100	6.6	18620.3	1316.2
WT527 APAP	TRXR1/GAPDH	C1:15	3	TRXR1	518	49	34006.6	464138	15.8	18.8	12.8	502.8	1267.2
WT527 APAP	TRXR1/GAPDH	C1:15	1	GAPDH	471	37	340991.6	2470730	100	100	6.8	5721.6	1150.3
WT528 APAP	TRXR1/GAPDH	C1:7	2	TRXR1	535	55	155645	1793764	17.2	20.7	10.8	2525.7	1918
WT528 APAP	TRXR1/GAPDH	C1:7	1	GAPDH	488	39	1115699	8654119	82.8	100	7.3	19804.3	1771.1
Alkbh8^{Def}546-/- APAP	TRXR1/GAPDH	C1:9	3	TRXR1	533	55	108093.9	1286538	16.1	19.1	11.2	1628.7	1461.1
Alkbh8^{Def}546-/- APAP	TRXR1/GAPDH	C1:10	2	GAPDH	491	38	339383.5	2344830	84.9	100	6.5	5840.2	1420.1
Alkbh8^{Def}547-/- APAP	TRXR1/GAPDH	C1:15	1	GAPDH	470	40	928417.5	7122660	100	100	7.2	13026.5	1735.4
Alkbh8^{Def}547-/- APAP	TRXR1/GAPDH	C1:15	3	TRXR1	516	55	87432.9	1292238	15.4	18.1	13.9	987.7	1780
Alkbh8^{Def}548-/- APAP	TRXR1/GAPDH	C1:6	1	GAPDH	487	39	1065369	7837010	85.5	100	6.9	17383.8	1631.5
Alkbh8^{Def}548-/- APAP	TRXR1/GAPDH	C1:6	2	TRXR1	534	55	118215.5	1324038	14.5	16.9	10.5	1797.2	1762.6
WT521Saline	TRXR2/GAPDH	C1:14	1	GAPDH	491	40	594262.4	3922895	97.7	100	6.2	48712.9	233.5
WT521Saline	TRXR2/GAPDH	C1:14	2	TRXR2	540	57	9670.1	94366	2.3	2.4	9.2	802.1	272.2
WT522Saline	TRXR2/GAPDH	C1:15	1	GAPDH	493	40	402909.6	2553664	97.3	100	6	25460	236.7
WT522Saline	TRXR2/GAPDH	C1:15	2	TRXR2	541	57	7146.2	70365	2.7	2.8	9.3	467	275.1
WT526Saline	TRXR2/GAPDH	C1:16	1	GAPDH	491	40	538677.9	3566008	98.1	100	6.2	48026.6	196.4
WT526Saline	TRXR2/GAPDH	C1:16	2	TRXR2	540	57	7411.7	69997	1.9	2	8.9	664.1	219.6
Alkbh8^{Def}549-/- Saline	TRXR2/GAPDH	C1:17	1	GAPDH	492	39	607590.5	4029011	97.7	100	6.2	52912.8	201.4
Alkbh8^{Def}549-/- Saline	TRXR2/GAPDH	C1:17	2	TRXR2	540	56	9377.1	93718	2.3	2.3	9.4	814.4	227.5
Alkbh8^{Def}550-/- Saline	TRXR2/GAPDH	C1:18	1	GAPDH	491	39	741488	5124450	97.5	100	6.5	73514.3	162.8
Alkbh8^{Def}550-/- Saline	TRXR2/GAPDH	C1:18	2	TRXR2	540	56	12812.5	129741	2.5	2.5	9.5	1262.6	201.6
Alkbh8^{Def}551-/- Saline	TRXR2/GAPDH	C1:19	1	GAPDH	491	39	703891.8	5230940	97.1	100	7	61262.2	177.6
Alkbh8^{Def}551-/- Saline	TRXR2/GAPDH	C1:19	2	TRXR2	540	56	13317.5	157850	2.9	3	11.1	1054.9	192.5
WT525 APAP	TRXR2/GAPDH	C1:18	2	GAPDH	469	39	517747.7	3643415	100	100	6.6	38919.5	260.8
WT525 APAP	TRXR2/GAPDH	C1:18	4	TRXR2	518	56	16681	238889	6.2	6.6	13.5	984.5	339.6
WT527 APAP	TRXR2/GAPDH	C1:21	2	GAPDH	492	40	575883.6	3989174	94.7	100	6.5	45127.9	154.9
WT527 APAP	TRXR2/GAPDH	C1:21	3	TRXR2	539	56	18934	223374	5.3	5.6	11.1	1387.8	189.8
WT528 APAP	TRXR2/GAPDH	C1:22	2	GAPDH	495	38	176883	1197732	93.2	100	6.4	15283.7	95.7

WT528 APAP	TRXR2/GAPDH	C1:22	3	TRXR2	543	51	7242.3	87202	6.8	7.3	11.3	616.3	103.6
Alkbh8^{Def}546-/- APAP	TRXR2/GAPDH	C1:23	1	GAPDH	493	40	685699.6	4875224	97	100	6.7	60851	102.7
Alkbh8^{Def}546-/- APAP	TRXR2/GAPDH	C1:23	2	TRXR2	541	56	13380.2	150990	3	3.1	10.6	1105.9	105.4
Alkbh8^{Def}547-/- APAP	TRXR2/GAPDH	C1:24	1	GAPDH	494	39	791208.9	5630355	96.6	100	6.7	68963.9	100.1
Alkbh8^{Def}547-/- APAP	TRXR2/GAPDH	C1:24	2	TRXR2	541	56	17871.9	199552	3.4	3.5	10.5	1488.4	107.5
Alkbh8^{Def}548-/- APAP	TRXR2/GAPDH	C1:25	2	GAPDH	495	39	882530.7	6432035	95.1	100	6.8	97145.7	107.8
Alkbh8^{Def}548-/- APAP	TRXR2/GAPDH	C1:25	3	TRXR2	542	55	28451.6	331057	4.9	5.1	10.9	2872.4	128.6
WT521Saline	SELS/GAPDH	C1:2	1	SELS	455	32	37948.4	395404	13.4	15.5	9.8	4425.3	91.2
WT521Saline	SELS/GAPDH	C1:2	2	GAPDH	487	40	383116.7	2544680	86.6	100	6.2	44452.3	111.6
WT522Saline	SELS/GAPDH	C1:4	1	SELS	453	32	45598.6	508807	21.2	26.9	10.5	3477.6	146.3
WT522Saline	SELS/GAPDH	C1:4	2	GAPDH	485	40	281968.7	1888015	78.8	100	6.3	21750.2	168
WT526Saline	SELS/GAPDH	C1:23	2	SELS	443	31	51349.6	631775	20.1	25.2	11.6	6799	55.5
WT526Saline	SELS/GAPDH	C1:23	3	GAPDH	473	37	298015.8	2509593	100	100	7.9	43284.7	58.3
Alkbh8^{Def}*549*-/- Saline	SELS/GAPDH	C1:5	1	SELS	451	32	39733.1	462656	18.8	23.1	10.9	3015.6	230.8
Alkbh8^{Def}*549*-/- Saline	SELS/GAPDH	C1:5	2	GAPDH	484	40	302264.8	2002088	81.2	100	6.2	23297.2	282.2
Alkbh8^{Def}*550*-/- Saline	SELS/GAPDH	C1:6	1	SELS	452	32	48520.1	535058	15.7	18.6	10.4	4049.7	145.6
Alkbh8^{Def}*550*-/- Saline	SELS/GAPDH	C1:6	2	GAPDH	485	40	440255.3	2870522	84.3	100	6.1	36845.8	168.7
Alkbh8^{Def}*551*-/- Saline	SELS/GAPDH	C1:7	1	SELS	454	32	59260.3	643864	16.1	19.2	10.2	3995.4	186.8
Alkbh8^{Def}*551*-/- Saline	SELS/GAPDH	C1:7	2	GAPDH	485	39	493997	3354100	83.9	100	6.4	33606.9	197.1
WT525 APAP	SELS/GAPDH	C1:8	1	SELS	454	32	41668.2	442586	15.1	17.8	10	3172.5	186.7
WT525 APAP	SELS/GAPDH	C1:8	2	GAPDH	486	40	378733.1	2480379	84.9	100	6.2	28855.3	215.8
WT527 APAP	SELS/GAPDH	C1:9	2	SELS	454	33	46856.7	513654	18.7	22.9	10.3	3615.9	153.5
WT527 APAP	SELS/GAPDH	C1:9	3	GAPDH	486	40	352122.7	2239899	81.3	100	6	27171.7	163.3
WT528 APAP	SELS/GAPDH	C1:11	1	SELS	456	32	58019.6	599189	17.6	21.3	9.7	3867.8	166.6
WT528 APAP	SELS/GAPDH	C1:11	2	GAPDH	487	40	442481.5	2814227	82.4	100	6	28843.3	170.6
Alkbh8^{Def}546-/- APAP	SELS/GAPDH	C1:25	2	SELS	446	31	68843.4	862059	33.1	49.4	11.8	6892.2	36.1
Alkbh8^{Def}546-/- APAP	SELS/GAPDH	C1:25	3	GAPDH	476	37	215157.8	1744480	100	100	7.6	24206	38
Alkbh8^{Def}547-/- APAP	SELS/GAPDH	C1:3	1	SELS	455	32	40197.2	455044	22.8	29.6	10.6	3017.8	104.9
Alkbh8^{Def}547-/- APAP	SELS/GAPDH	C1:3	2	GAPDH	487	40	222988	1539343	77.2	100	6.5	17058	118.6
Alkbh8^{Def}548-/- APAP	SELS/GAPDH	C1:10	1	SELS	456	32	28508.3	322789	37.3	59.4	10.6	1705.3	151.9
Alkbh8^{Def}548-/- APAP	SELS/GAPDH	C1:10	2	GAPDH	489	38	81626.7	543644	62.7	100	6.3	4941.9	158.7

Supplemental Table S3. WES raw data for all proteins analyzed in 4 day APAP exposure experiment. Protein quantitation data was normalized to housekeeping protein, GAPDH, and normalized corrected area analysis setting was set to 100 on ProteinSimple Compass Software.

Sample	Primary Antibody	Capillary	Peak	Name	Position	MW (kDa)	Height	Area	% Area	Corr. Area	Width	S/N	Baseline
WT31Saline	GPX1/GAPDH	C1:2	1	GPX1	418	28	40129.5	343312	18.2	22.3	8	1340.9	259.9
WT31Saline	GPX1/GAPDH	C1:2	2	GAPDH	466	39	233098.3	1541883	100	100	6.2	7363.4	286.6
WT32Saline	GPX1/GAPDH	C1:3	1	GPX1	415	28	35244.6	298654	21.3	27	8	1032.7	282.7
WT32Saline	GPX1/GAPDH	C1:3	2	GAPDH	463	39	173813.4	1105148	100	100	6	4664	347.1
WT33Saline	GPX1/GAPDH	C1:4	1	GPX1	413	28	74896.2	536060	19.4	24.1	6.7	2075.8	378.9
WT33Saline	GPX1/GAPDH	C1:4	2	GAPDH	460	39	350630.2	2224005	100	100	6	9321.9	428.4
*Alkbh8^{Def}*385-/- Saline	GPX1/GAPDH	C1:24	1	GPX1	429	28	86820.3	689265	5.7	6.1	7.5	2393.5	382.5
*Alkbh8^{Def}*385-/- Saline	GPX1/GAPDH	C1:24	3	GAPDH	481	39	1358158	11369844	93.9	100	7.9	41919.1	435.9
*Alkbh8^{Def}*33-/- Saline	GPX1/GAPDH	C1:13	1	GPX1	417	28	25332.9	184464	5	5.3	6.8	1410.2	334.5
*Alkbh8^{Def}*33-/- Saline	GPX1/GAPDH	C1:13	2	GAPDH	467	39	547989.3	3493769	100	100	6	30166.5	387.6
*Alkbh8^{Def}*31-/- Saline	GPX1/GAPDH	C1:6	1	GPX1	413	28	11044	101371	3.9	4.1	8.6	475.8	395.4
*Alkbh8^{Def}*31-/- Saline	GPX1/GAPDH	C1:6	2	GAPDH	464	39	371041.7	2471133	100	100	6.3	14955.4	490.1
WT84 APAP	GPX1/GAPDH	C1:4	2	GPX1	417	28	32896.5	262136		48.6	7.5	612.6	721.5
WT84 APAP	GPX1/GAPDH	C1:4	3	GAPDH	465	40	88959.1	539041	89.3	100	5.7	1494.6	795.9
WT33 APAP	GPX1/GAPDH	C1:7	1	GPX1	417	28	26481.2	212835	19	23.4	7.6	923.4	521.6
WT33 APAP	GPX1/GAPDH	C1:7	2	GAPDH	463	39	140183.8	909708	100	100	6.1	4588.3	739.1
WT550 APAP	GPX1/GAPDH	C1:17	4	GPX1	418	28	32929.6	480163	24.4	32.4	13.7	461.5	1399.6
WT550 APAP	GPX1/GAPDH	C1:17	2	GAPDH	472	39	199006.1	1484222	100	100	7	3215.4	1279.5
*Alkbh8^{Def}*30-/- APAP	GPX1/GAPDH	C1:11	1	GPX1	418	28	20830	163081	9.9	11	7.4	566.2	459.9
*Alkbh8^{Def}*30-/- APAP	GPX1/GAPDH	C1:11	2	GAPDH	465	39	247986.2	1482107	100	100	5.6	6230.3	506
*Alkbh8^{Def}*31-/- APAP	GPX1/GAPDH	C1:12	1	GPX1	416	28	24618.2	183857	8.6	9.4	7	645.9	467.4
*Alkbh8^{Def}*31-/- APAP	GPX1/GAPDH	C1:12	2	GAPDH	463	39	339718.5	1947598	100	100	5.4	8081.1	518.4
*Alkbh8^{Def}*33-/- APAP	GPX1/GAPDH	C1:13	1	GPX1	418	28	44700.6	335308	11.4	12.8	7	942.9	567.2
*Alkbh8^{Def}*33-/- APAP	GPX1/GAPDH	C1:13	2	GAPDH	464	39	419842.9	2610890	100	100	5.8	8473.6	601.6
WT31Saline	GPX3/GAPDH	C1:14	1	GPX3	417	28	2225.4	19920	1.2	1.2	8.4	44.3	461.5
WT31Saline	GPX3/GAPDH	C1:14	2	GAPDH	465	39	267941.1	1670047	100	100	5.9	6192	529.1

WT32Saline	GPX3/GAPDH	C1:15	1	GPX3	418	28	1871.1	16611	1.1	1.1	8.3	43.7	557.2
WT32Saline	GPX3/GAPDH	C1:15	2	GAPDH	464	39	245808.4	1541493	100	100	5.9	6146.2	650.1
WT33Saline	GPX3/GAPDH	C1:16	1	GPX3	417	28	6493.9	51461	2	2	7.4	190.6	408
WT33Saline	GPX3/GAPDH	C1:16	2	GAPDH	462	39	410524.9	2582026	100	100	5.9	12116.8	415.4
Alkbh8^{Def}385-/- Saline	GPX3/GAPDH	C1:17	1	GPX3	419	28	2850.7	22703	1.3	1.3	7.5	60.3	449.1
Alkbh8^{Def}385-/- Saline	GPX3/GAPDH	C1:17	2	GAPDH	465	39	287822	1759182	100	100	5.7	6858.2	482.6
Alkbh8^{Def}33-/- Saline	GPX3/GAPDH	C1:18	1	GPX3	420	28	4080.6	30441	1.3	1.3	7	126.8	503
Alkbh8^{Def}33-/- Saline	GPX3/GAPDH	C1:18	2	GAPDH	466	39	364356.7	2306516	100	100	5.9	11428.3	576.2
Alkbh8^{Def}31-/- Saline	GPX3/GAPDH	C1:19	1	GPX3	418	28	1612.5	12533	0.7	0.7	7.3	43.9	644.8
Alkbh8^{Def}31-/- Saline	GPX3/GAPDH	C1:19	2	GAPDH	465	39	271618	1789541	100	100	6.2	7696.1	915.3
WT84 APAP	GPX3/GAPDH	C1:2	1	GPX4	415	27	11639.5	102347	3.9	4	8.3	437.4	287.2
WT84 APAP	GPX3/GAPDH	C1:2	2	GAPDH	467	39	397058.3	2539391	100	100	6	13927	368.7
WT33 APAP	GPX3/GAPDH	C1:23	1	GPX3	452	28	5868.4	57576	2	2	9.2	138	475.7
WT33 APAP	GPX3/GAPDH	C1:23	2	GAPDH	494	38	337697.6	2856441	98	100	7.9	8020.9	557.2
WT550 APAP	GPX3/GAPDH	C1:22	1	GPX3	455	28	1819	20147	4.5	4.7	10.4	50.5	489.3
WT550 APAP	GPX3/GAPDH	C1:22	2	GAPDH	500	37	55633.7	431484	95.5	100	7.3	1716.3	627.1
Alkbh8^{Def}30-/- APAP	GPX3/GAPDH	C1:20	1	GPX3	418	28	1131.4	10178	0.5	0.5	8.5	17.9	539.5
Alkbh8^{Def}30-/- APAP	GPX3/GAPDH	C1:20	2	GAPDH	465	39	324220.5	2075199	100	100	6	7306.2	639.7
Alkbh8^{Def}31-/- APAP	GPX3/GAPDH	C1:23	1	GPX3	418	28	1024	9055	0.4	0.4	8.3	19.6	537.9
Alkbh8^{Def}31-/- APAP	GPX3/GAPDH	C1:23	2	GAPDH	466	39	310289.9	2017076	100	100	6.1	7838.1	621.1
Alkbh8^{Def}33-/- APAP	GPX3/GAPDH	C1:24	1	GPX3	421	28	2820.5	22825	1.2	1.2	7.6	79.2	380.5
Alkbh8^{Def}33-/- APAP	GPX3/GAPDH	C1:24	2	GAPDH	466	39	293863.9	1863307	100	100	6	8991.1	397.6
WT31Saline	GPX4/GAPDH	C1:6	1	GPX4	429	27	35695.8	300060	5	5.2	7.9	1537.5	388.6
WT31Saline	GPX4/GAPDH	C1:6	2	GAPDH	486	39	853578.7	5741046	95	100	6.3	36274.7	464.3
WT32Saline	GPX4/GAPDH	C1:22	1	GPX4	426	27	213285.6	1648104	10	11.2	7.3	6894.5	429.2
WT32Saline	GPX4/GAPDH	C1:22	3	GAPDH	475	39	1414955	14734174	89.2	100	9.8	50726.5	504.7
WT33Saline	GPX4/GAPDH	C1:23	1	GPX4	427	27	144364.1	1132737	8.1	8.9	7.4	3911	388.8
WT33Saline	GPX4/GAPDH	C1:23	3	GAPDH	479	40	1403219	12712120	91	100	8.5	42555.5	456.2
Alkbh8^{Def}385-/- Saline	GPX4/GAPDH	C1:2	1	GPX4	415	27	11639.5	102347	3.9	4	8.3	437.4	287.2

Alkbh8^{Def}385-/- Saline	GPX4/GAPDH	C1:2	2	GAPDH	467	39	397058.3	2539391	100	100	6	13927	368.7
Alkbh8^{Def}33-/- Saline	GPX4/GAPDH	C1:3	1	GPX4	415	27	9348.2	86532	4.5	4.7	8.7	433.6	420.8
Alkbh8^{Def}33-/- Saline	GPX4/GAPDH	C1:3	2	GAPDH	467	39	266535.3	1835188	100	100	6.5	12001.4	624.8
Alkbh8^{Def}31-/- Saline	GPX4/GAPDH	C1:6	1	GPX4	413	27	11044	101371	3.9	4.1	8.6	475.8	395.4
Alkbh8^{Def}31-/- Saline	GPX4/GAPDH	C1:6	2	GAPDH	464	39	371041.7	2471133	100	100	6.3	14955.4	490.1
WT84 APAP	GPX4/GAPDH	C1:5	1	GPX4	421	27	202436.9	1583407	9.1	10.1	7.3	5175.4	441.1
WT84 APAP	GPX4/GAPDH	C1:5	2	GAPDH	470	39	1532362	15748273	90.9	100	9.7	43306.9	501.1
WT33 APAP	GPX4/GAPDH	C1:6	1	GPX4	422	27	215369.8	1621761	9.3	10.2	7.1	3960.5	574.6
WT33 APAP	GPX4/GAPDH	C1:6	2	GAPDH	470	39	1516815	15896473	90.7	100	9.8	30523.8	675.2
WT550 APAP	GPX4/GAPDH	C1:24	1	GPX4	429	27	86820.3	689265	5.7	6.1	7.5	2393.5	382.5
WT550 APAP	GPX4/GAPDH	C1:24	3	GAPDH	481	41	1358158	11369844	93.9	100	7.9	41919.1	435.9
Alkbh8^{Def}30-/- APAP	GPX4/GAPDH	C1:8	1	GPX4	416	27	11443	108125	4.1	4.3	8.9	542.1	321.1
Alkbh8^{Def}30-/- APAP	GPX4/GAPDH	C1:8	2	GAPDH	468	40	388739.6	2538523	100	100	6.1	17065.4	399.6
Alkbh8^{Def}31-/- APAP	GPX4/GAPDH	C1:9	1	GPX4	415	27	20099.5	156261	4.2	4.4	7.3	989.6	362.6
Alkbh8^{Def}31-/- APAP	GPX4/GAPDH	C1:9	2	GAPDH	465	39	531114.3	3532639	100	100	6.2	26046.1	415.5
Alkbh8^{Def}33-/- APAP	GPX4/GAPDH	C1:11	1	GPX4	416	27	10104.2	86419	4.7	4.9	8	428.1	496.9
Alkbh8^{Def}33-/- APAP	GPX4/GAPDH	C1:11	2	GAPDH	468	39	282969.1	1746631	100	100	5.8	10899.3	665.7
WT31Saline	TRXR1/GAPDH	C1:14	3	TRXR1	514	56	70222.4	759048	29.3	41.4	10.2	991.3	1416.8
WT31Saline	TRXR1/GAPDH	C1:14	1	GAPDH	469	39	288439.9	1834181	100	100	6	4035.9	1370.5
WT32Saline	TRXR1/GAPDH	C1:15	4	TRXR1	512	55	57290.6	628364	31.5	46.1	10.3	800.1	1275.9
WT32Saline	TRXR1/GAPDH	C1:15	1	GAPDH	468	39	210057.9	1364096	100	100	6.1	3018.2	1245
WT33Saline	TRXR1/GAPDH	C1:16	3	TRXR1	513	55	91597.6	1160188	25.4	34.1	11.9	1126.5	1348.1
WT33Saline	TRXR1/GAPDH	C1:16	1	GAPDH	468	39	529470.9	3402601	100	100	6	6936.3	1285.6
Alkbh8^{Def}385-/- Saline	TRXR1/GAPDH	C1:7	1	GAPDH	449	39	1192115	10935916	85	100	8.6	27842.1	1347.8
Alkbh8^{Def}385-/- Saline	TRXR1/GAPDH	C1:7	2	TrxR1	496	55	115887.5	1930032	15	17.6	15.6	1842.9	1504.2
Alkbh8^{Def}33-/- Saline	TRXR1/GAPDH	C1:8	2	GAPDH	452	40	1143360	9046184	86.1	100	7.4	19409.5	1468.8

Alkbh8^{Def}33-/- Saline	TRXR1/GAPDH	C1:8	3	TrxR1	497	55	104298.7	1465583	13.9	16.2	13.2	1410.2	1658
Alkbh8^{Def}31-/- Saline	TRXR1/GAPDH	C1:10	2	GAPDH	452	40	1191875	9877595	86.7	100	7.8	21024.1	1153.4
Alkbh8^{Def}31-/- Saline	TRXR1/GAPDH	C1:10	3	TrxR1	499	55	114268.8	1521465	13.3	15.4	12.5	1654.9	1226.7
WT84 APAP	TRXR1/GAPDH	C1:23	1	GAPDH	468	39	337495.2	2140557	100	100	6	7736.6	1102.1
WT84 APAP	TRXR1/GAPDH	C1:23	3	TRXR1	512	55	157877.7	1693620	44.2	79.1	10.1	3738.4	1138.2
WT33 APAP	TRXR1/GAPDH	C1:24	1	GAPDH	469	39	532928.8	3489732	100	100	6.2	9395.7	1315.3
WT33 APAP	TRXR1/GAPDH	C1:24	3	TRXR1	513	55	222038.5	2540597	42.1	72.8	10.7	3868.5	1440.9
WT550 APAP	TRXR1/GAPDH	C1:25	1	GAPDH	470	39	657591.1	4431630	100	100	6.3	13510.2	1108
WT550 APAP	TRXR1/GAPDH	C1:25	3	TRXR1	513	55	228184.6	2886554	39.4	65.1	11.9	4270.2	1093.2
Alkbh8^{Def}30-/- APAP	TRXR1/GAPDH	C1:20	1	GAPDH	469	39	446080.5	2893694	100	100	6.1	7801.8	1127.3
Alkbh8^{Def}30-/- APAP	TRXR1/GAPDH	C1:20	3	TRXR1	513	55	123676.9	1312675	31.2	45.4	10	2212.4	1182.3
Alkbh8^{Def}31-/- APAP	TRXR1/GAPDH	C1:11	3	GAPDH	471	39	1399523	14786023	72.7	100	9.9	30745.4	1509.4
Alkbh8^{Def}31-/- APAP	TRXR1/GAPDH	C1:11	4	TrxR1	517	55	384227.8	5559228	27.3	37.6	13.6	6450.2	1620.5
Alkbh8^{Def}33-/- APAP	TRXR1/GAPDH	C1:12	2	GAPDH	472	39	1365379	14166739	77.5	100	9.7	28320.6	1507.5
Alkbh8^{Def}33-/- APAP	TRXR1/GAPDH	C1:12	3	TrxR1	518	54	261367.2	4098671	22.4	28.9	14.7	3891.8	1599.7
WT31Saline	TRXR2/GAPDH	C1:2	3	GAPDH	464	39	459326.5	3055865	100	100	6.3	53915.9	95.3
WT31Saline	TRXR2/GAPDH	C1:2	5	TRXR2	511	56	14701.9	159784	5	5.2	10.2	1650.1	107.1
WT32Saline	TRXR2/GAPDH	C1:3	2	GAPDH	466	41	138965.2	803676		100	5.4	22106.5	81
WT32Saline	TRXR2/GAPDH	C1:3	3	TRXR2	513	57	4983.4	39637	100	4.9	7.5	825	91.5
WT33Saline	TRXR2/GAPDH	C1:4	2	GAPDH	466	40	511223.5	3391163	100	100	6.2	75772.4	104.5
WT33Saline	TRXR2/GAPDH	C1:4	4	TRXR2	513	56	13117.2	110304	3.2	3.3	7.9	1680.6	111.5
Alkbh8^{Def}385-/- Saline	TRXR2/GAPDH	C1:5	1	GAPDH	467	40	184022.2	1131687	100	100	5.8	16344.3	129.4
Alkbh8^{Def}385-/- Saline	TRXR2/GAPDH	C1:5	3	TRXR2	513	57	4608.7	45131	3.8	4	9.2	430.7	139.1
Alkbh8^{Def}33-/- Saline	TRXR2/GAPDH	C1:6	1	GAPDH	468	40	199282.1	1205769	100	100	5.7	21475	141.5

Alkbh8^{Def}33-/- Saline	TRXR2/GAPDH	C1:6	3	TRXR2	514	57	5630.6	43180	3.5	3.6	7.2	606.9	150.9
Alkbh8^{Def}31-/- Saline	TRXR2/GAPDH	C1:7	1	GAPDH	468	40	113837.9	659408	100	100	5.4	13882.6	125
Alkbh8^{Def}31-/- Saline	TRXR2/GAPDH	C1:7	3	TRXR2	515	57	4228.7	33727	4.9	5.1	7.5	546.2	136.9
WT84 APAP	TRXR2/GAPDH	C1:10	2	GAPDH	455	40	560704.7	3692772	93.8	100	6.2	126044	107.1
WT84 APAP	TRXR2/GAPDH	C1:10	4	TRXR2	505	56	22075.1	243972	6.2	6.6	10.4	4803.3	109.6
WT33 APAP	TRXR2/GAPDH	C1:7	2	GAPDH	445	38	1148180	9860372	100	99.4	8.1	185483.9	72.1
WT33 APAP	TRXR2/GAPDH	C1:7	4	TRXR2	496	55	40732	571183	5.5	5.8	13.2	4990.5	79.2
WT550 APAP	TRXR2/GAPDH	C1:9	2	GAPDH	446	38	1034947	8687592	100	99.5	7.9	200147.3	46.2
WT550 APAP	TRXR2/GAPDH	C1:9	4	TRXR2	496	55	42693.7	573814	6.2	6.6	12.6	6771.6	47.3
Alkbh8^{Def}30-/- APAP	TRXR2/GAPDH	C1:2	3	GAPDH	464	39	459326.5	3055865	100	100	6.3	53915.9	95.3
Alkbh8^{Def}30-/- APAP	TRXR2/GAPDH	C1:2	5	TRXR2	511	56	14701.9	159784	5	5.2	10.2	1650.1	107.1
Alkbh8^{Def}31-/- APAP	TRXR2/GAPDH	C1:4	2	GAPDH	466	40	511223.5	3391163	100	100	6.2	75772.4	104.5
Alkbh8^{Def}31-/- APAP	TRXR2/GAPDH	C1:4	4	TRXR2	513	56	13117.2	110304	3.2	3.3	7.9	1680.6	111.5
Alkbh8^{Def}33-/- APAP	TRXR2/GAPDH	C1:7	1	GAPDH	468	40	113837.9	659408	100	100	5.4	13882.6	125
Alkbh8^{Def}33-/- APAP	TRXR2/GAPDH	C1:7	3	TRXR2	515	57	4228.7	33727	4.9	5.1	7.5	546.2	136.9
WT31Saline	SELS/GAPDH	C1:4	2	SELS	421	32	79261.9	922410	14.1	16.4	10.9	8359.1	103
WT31Saline	SELS/GAPDH	C1:4	3	GAPDH	453	39	779181.8	5624588	99.2	100	6.8	88777.5	109.8
WT32Saline	SELS/GAPDH	C1:16	1	SELS	436	32	29157.4	288728	12.5	14.2	9.3	2931.7	156.3
WT32Saline	SELS/GAPDH	C1:16	2	GAPDH	469	40	342267.1	2027123	100	100	5.6	32854.4	160.3
WT33Saline	SELS/GAPDH	C1:23	1	SELS	436	32	30310.8	290941	15.5	18.3	9	2488.5	109.3
WT33Saline	SELS/GAPDH	C1:23	2	GAPDH	469	40	264143.3	1587239	100	100	5.6	20216.5	110.3
Alkbh8^{Def}385-/- Saline	SELS/GAPDH	C1:17	1	SELS	434	32	30123.1	301807	18	21.9	9.4	3740.7	135.9
Alkbh8^{Def}385-/- Saline	SELS/GAPDH	C1:17	2	GAPDH	467	40	231362	1378443	100	100	5.6	27341.6	137.6
Alkbh8^{Def}33-/- Saline	SELS/GAPDH	C1:18	1	SELS	435	32	27300.9	257911	17.8	21.7	8.9	2525.7	130.9
Alkbh8^{Def}33-/- Saline	SELS/GAPDH	C1:18	2	GAPDH	469	40	210540.7	1187568	100	100	5.3	17658.2	133.3
Alkbh8^{Def}31-/- Saline	SELS/GAPDH	C1:19	1	SELS	436	32	15532.7	157809	100	20.1	9.5	1124.4	242.3

Alkbh8^{Def}31-/- Saline	SELS/GAPDH	C1:19	3	GAPDH	470	41	133765.8	786573	100	100	5.5	8907.7	271.1
WT84 APAP	SELS/GAPDH	C1:20	1	SELS	437	32	30193.8	277491	100	17.1	8.6	4104.3	83.4
WT84 APAP	SELS/GAPDH	C1:20	3	GAPDH	470	40	275985.2	1622128	100	100	5.5	34281.3	84.2
WT33 APAP	SELS/GAPDH	C1:21	2	SELS	438	32	45701.1	431390	17.1	20.7	8.9	4895.7	95.5
WT33 APAP	SELS/GAPDH	C1:21	3	GAPDH	470	40	340743.1	2085712	100	100	5.8	33949.3	94.9
WT550 APAP	SELS/GAPDH	C1:25	1	SELS	437	32	62549.1	592575	19.4	24.1	8.9	5819	92.4
WT550 APAP	SELS/GAPDH	C1:25	2	GAPDH	469	40	401714.2	2456859	100	100	5.7	35401.9	99.8
Alkbh8^{Def}30-/- APAP	SELS/GAPDH	C1:24	1	SELS	435	32	62576.1	600118	30.4	43.8	9	7092.5	100
Alkbh8^{Def}30-/- APAP	SELS/GAPDH	C1:24	2	GAPDH	468	40	231594	1371013	100	100	5.6	24224.9	107.4
Alkbh8^{Def}31-/- APAP	SELS/GAPDH	C1:9	2	SELS	459	32	3390.9	31201	41.1	44.1	8.6	1073.4	66.6
Alkbh8^{Def}31-/- APAP	SELS/GAPDH	C1:9	4	GAPDH	493	41	9556.5	70728	100	100	7	3074.8	67.5
Alkbh8^{Def}33-/- APAP	SELS/GAPDH	C1:11	3	SELS	455	32	11024.4	121213	32.5	32.9	10.3	2213.3	67
Alkbh8^{Def}33-/- APAP	SELS/GAPDH	C1:11	5	GAPDH	488	40	54613.2	368957	100	100	6.3	11025.6	65.1

Supplemental Table S41. All measured epitranscriptomic marks after daily 600 mg/kg APAP for 4 days. Calculations for each epitranscriptomic gene count and comparisons between WT and Alkbh8^{Def} liver tissue post 4 day exposure to 600 mg/kg of APAP with reported statistical significance of biological replicates (N = 3) measured by an unpaired t-test. Increased expression (> 0) is reported in red shade and gene counts expressed (< 0) were reported shaded green. Significant comparisons (p ≤ 0.05) are shaded in yellow.

	WT Saline Average	*Alkbh8^{Def} Saline Average*	WT APAP Average	*Alkbh8^{Def} APAP Average*	WT APAP vs. WT Saline p-Value	*Alkbh8^{Def}* Saline vs WT Saline p-Value	*Alkbh8^{Def}* APAP vs. WT APAP p-Value	*Alkbh8^{Def}* APAP vs. *Alkbh8^{Def}* Saline p-Value	WT APAP vs. WT Saline, change in expression	*Alkbh8^{Def}* APAP vs. *Alkbh8^{Def}* Saline, change in expression	*Alkbh8^{Def}* Saline vs WT Saline, change in expression	*Alkbh8^{Def}* APAP vs. WT APAP, change in expression
Alkbh1	73.98	78.98	67.82	98.30	0.38617	0.41215	0.05893	0.15855	-6.16	19.32	5.01	30.48
Alkbh2	52.00	28.86	30.29	22.65	0.0152	0.00014	0.17617	0.07932	-21.71	-6.22	-23.14	-7.64
Alkbh3	147.78	159.45	148.05	135.45	0.49624	0.3115	0.30684	0.12311	0.27	-24.01	11.67	-12.60
Alkbh4	49.71	39.23	55.95	35.70	0.28697	0.06076	0.05927	0.27426	6.23	-3.52	-10.49	-20.24
Alkbh5	1130.44	1038.18	808.50	758.99	0.03791	0.23968	0.27428	0.00095	-321.94	-279.18	-92.27	-49.51
Alkbh6	99.37	76.45	101.22	81.48	0.46917	0.10266	0.19455	0.34762	1.85	5.03	-22.92	-19.74
Alkbh7	174.01	151.43	160.68	100.26	0.4053	0.32116	0.10981	0.09399	-13.33	-51.17	-22.58	-60.42
Alkbh8	82.01	38.30	85.23	31.02	0.37806	0.00165	0.00264	0.18383	3.22	-7.28	-43.72	-54.21
Alyref(Reader)	141.61	122.32	107.54	170.92	0.03746	0.09447	0.1152	0.16681	-34.08	48.60	-19.29	63.39
Cdk5rap1	129.16	183.84	92.11	96.32	0.04749	0.06048	0.40509	0.01664	-37.05	-87.53	54.68	4.20
Cdkal1	85.95	83.04	57.15	75.42	0.00755	0.374	0.0575	0.24849	-28.80	-7.62	-2.91	18.27
Ctu1	59.18	64.95	59.03	46.69	0.4933	0.1007	0.15062	0.0355	-0.15	-18.26	5.77	-12.34
Ctu2	143.27	111.51	92.14	116.24	0.07533	0.14326	0.18422	0.41275	-51.14	4.73	-31.76	24.11
Dus2	88.19	105.12	119.38	101.36	0.10431	0.26561	0.07961	0.41745	31.19	-3.77	16.93	-18.03
Elp1	110.06	118.77	130.22	189.31	0.14598	0.30582	0.04925	0.02986	20.15	70.55	8.70	59.10
Elp2	325.37	342.29	314.51	361.74	0.37353	0.21089	0.11272	0.20728	-10.86	19.46	16.91	47.23
Elp3	304.85	301.35	290.18	297.63	0.28651	0.3805	0.43521	0.4625	-14.66	-3.72	-3.50	7.45
Elp4	99.38	111.68	88.78	75.55	0.31457	0.31008	0.13255	0.03532	-10.60	-36.13	12.30	-13.23
Elp5	269.38	233.06	224.48	203.21	0.19405	0.10719	0.31927	0.0548	-44.89	-29.85	-36.32	-21.28
Elp6	69.04	46.65	71.10	43.73	0.46117	0.17065	0.05585	0.42666	2.06	-2.92	-22.40	-27.37
Fto	413.52	390.51	383.12	367.09	0.1863	0.24778	0.35434	0.2959	-30.40	-23.42	-23.01	-16.03
Ftsj	232.76	208.72	199.45	163.81	0.24157	0.21426	0.20631	0.04516	-33.30	-44.91	-24.04	-35.65
Gtpbp3	77.68	93.87	127.22	128.61	0.01746	0.07273	0.4676	0.01053	49.55	34.74	16.20	1.39
Lcmt2	103.47	84.42	118.72	88.80	0.12429	0.05285	0.03798	0.35122	15.24	4.38	-19.05	-29.91
Mettl1	203.79	129.68	154.39	137.72	0.28002	0.13156	0.39856	0.39913	-49.40	8.04	-74.11	-16.66
Mettl2	84.69	92.55	87.78	84.27	0.37661	0.05017	0.36447	0.06418	3.09	-8.28	7.85	-3.51
Mettl3	66.29	70.89	55.52	63.39	0.15684	0.27013	0.21433	0.15058	-10.76	-7.50	4.61	7.87
Mettl4	106.43	155.03	158.59	132.28	0.0166	0.00132	0.16838	0.15064	52.16	-22.76	48.60	-26.32

Metll5	118.34	136.94	100.12	112.50	0.1003	0.10149	0.09291	0.02085	-18.21	-24.44	18.60	12.38
Mettl6	147.33	128.24	115.69	145.65	0.03535	0.08898	0.0072	0.01032	-31.63	17.41	-19.09	29.95
Mettl7a	2870.64	2920.69	3248.80	2055.12	0.29183	0.45992	0.06752	0.07031	378.16	-865.58	50.05	-1193.69
Mettl7b	10592.57	6944.21	8639.57	3520.68	0.15229	0.00461	0.0409	0.05381	-1953.00	-3423.53	-3648.36	-5118.89
Metll8	139.37	145.35	140.80	91.27	0.46341	0.27833	0.07667	0.05218	1.43	-54.09	5.98	-49.53
Mettl9	550.19	516.06	479.71	479.98	0.08811	0.19757	0.49877	0.33681	-70.48	-36.08	-34.13	0.27
Mettl14	62.43	69.90	59.79	75.30	0.36533	0.25572	0.11849	0.35455	-2.64	5.40	7.48	15.51
Mettl15	19.82	37.34	35.27	51.22	0.06461	0.01622	0.07118	0.04764	15.44	13.88	17.52	15.96
Mettl16	161.87	171.95	197.76	146.28	0.01825	0.29355	0.06712	0.22161	35.89	-25.67	10.08	-51.48
Mettl17	123.08	135.74	159.27	113.11	0.01793	0.14895	0.05417	0.17967	36.19	-22.63	12.66	-46.16
Mettl18	37.42	24.97	37.05	36.95	0.46264	0.01138	0.49434	0.0752	-0.37	11.97	-12.45	-0.10
Mettl21a	81.33	85.59	101.19	110.50	0.01282	0.12383	0.15139	0.0082	19.86	24.91	4.26	9.31
Mettl21c	1.31	1.12	1.13	1.00	0.41118	0.36357	0.42827	0.38964	-0.18	-0.12	-0.19	-0.13
Mettl22	144.07	94.70	91.14	60.20	0.07132	0.04983	0.10819	0.02067	-52.93	-34.51	-49.36	-30.94
Mettl23	230.61	178.61	223.27	127.46	0.4608	0.17554	0.09191	0.09823	-7.34	-51.15	-52.00	-95.81
Mettl24	1.93	0.50	2.06	1.80	0.45367	0.08876	0.37217	0.02546	0.13	1.30	-1.43	-0.26
Mettl25	27.95	22.04	14.08	20.85	0.03402	0.14029	0.0699	0.30827	-13.88	-1.19	-5.91	6.77
Mettl26	2360.65	1646.13	1154.60	527.87	0.02172	0.0574	0.04817	0.0024	-1206.06	-1118.26	-714.53	-626.73
Mettl27	127.33	138.04	159.16	115.41	0.16902	0.34275	0.13318	0.24583	31.82	-22.63	10.70	-43.75
Mrm1	44.76	29.41	27.49	33.11	0.02882	0.01161	0.2796	0.31969	-17.26	3.70	-15.35	5.61
Mrm2	116.37	83.06	112.92	62.56	0.46134	0.08474	0.08852	0.1239	-3.45	-20.50	-33.31	-50.36
Mrm3	95.96	89.59	81.39	64.88	0.19694	0.33215	0.10529	0.02338	-14.57	-24.72	-6.37	-16.52
Mto1	176.24	204.54	142.39	115.38	0.02327	0.03939	0.04366	0.00092	-33.85	-89.16	28.30	-27.02
Nat10	170.18	201.57	175.69	245.04	0.37333	0.02541	0.00938	0.01922	5.52	43.47	31.39	69.35
Nsun2	917.83	927.88	702.01	817.25	0.03412	0.45256	0.18573	0.18318	-215.82	-110.63	10.05	115.24
Nsun3	108.87	144.98	119.67	125.89	0.32017	0.0517	0.36873	0.0885	10.81	-19.09	36.11	6.22
Nsun4	242.17	218.69	183.43	140.94	0.00379	0.08839	0.05045	0.01138	-58.74	-77.76	-23.47	-42.49
Osgepl1	122.29	127.99	119.15	113.54	0.43918	0.30001	0.39344	0.11881	-3.13	-14.45	5.70	-5.61
Pus1	207.13	168.55	183.54	153.50	0.26833	0.05879	0.21007	0.20756	-23.60	-15.05	-38.59	-30.04
Qtrt1	43.82	35.84	46.47	44.53	0.42115	0.26256	0.43654	0.22411	2.65	8.69	-7.99	-1.95
Qtrt2	49.80	60.79	37.74	73.04	0.15201	0.20697	0.0186	0.20232	-12.06	12.24	11.00	35.30
Tarbp1	41.44	66.44	40.93	66.71	0.48452	0.03032	0.02725	0.48116	-0.51	0.28	25.00	25.79
Thumpd1	227.25	230.09	230.30	269.92	0.44034	0.45497	0.07394	0.10112	3.05	39.82	2.84	39.61
Trdmt1	11.96	23.45	28.91	32.85	0.00518	0.01077	0.22076	0.04353	16.95	9.40	11.49	3.94
Trit1	174.82	158.85	142.30	114.92	0.12494	0.24201	0.1117	0.01889	-32.51	-43.93	-15.96	-27.38
Trmo	40.67	35.51	22.83	23.55	0.06921	0.30405	0.41931	0.00192	-17.84	-11.96	-5.16	0.72
Trmt1l	212.92	248.96	239.71	189.76	0.04962	0.03073	0.00633	0.00543	26.80	-59.20	36.04	-49.96

Trmt2a	132.89	148.63	143.77	207.62	0.27882	0.17184	0.03717	0.03948	10.88	58.99	15.74	63.85
Trmt5	62.21	77.99	58.25	73.23	0.34699	0.09043	0.15385	0.36724	-3.97	-4.76	15.78	14.98
Trmt6	165.95	145.60	142.59	227.47	0.23101	0.22666	0.08884	0.08759	-23.36	81.86	-20.35	84.88
Trmt9b	39.57	78.90	49.31	13.20	0.14461	0.06525	0.00397	0.01633	9.74	-65.70	39.33	-36.11
Trmt10a	48.85	64.08	63.78	57.23	0.06272	0.08796	0.33027	0.3332	14.93	-6.85	15.23	-6.55
Trmt10b	62.21	77.99	58.25	73.23	0.34699	0.09043	0.15385	0.36724	-3.97	-4.76	15.78	14.98
Trmt10c	212.30	220.26	133.66	183.68	0.00048	0.16597	0.00876	0.01726	-78.64	-36.58	7.96	50.02
Trmt11	61.24	65.98	45.32	59.65	0.02883	0.24307	0.05141	0.20616	-15.93	-6.34	4.74	14.33
Trmt12	34.34	47.12	77.26	57.32	0.01088	0.00882	0.09919	0.09165	42.92	10.19	12.79	-19.94
Trmt13	19.77	23.22	16.93	35.51	0.08531	0.26766	0.05227	0.14496	-2.84	12.30	3.44	18.58
Trmt61a	51.37	42.51	31.85	68.43	0.04394	0.14339	0.06035	0.11128	-19.52	25.92	-8.86	36.58
Trmt61b	15.03	24.19	15.49	26.56	0.44874	0.09342	0.03759	0.36848	0.47	2.36	9.16	11.06
Trmt112	221.23	155.83	159.95	103.52	0.08408	0.03662	0.04213	7.5E-05	-61.28	-52.31	-65.40	-56.43
Trmu	109.05	90.53	101.58	119.12	0.41799	0.23967	0.27837	0.04694	-7.47	28.59	-18.52	17.54
Trub2	64.75	88.24	74.50	107.28	0.18471	0.02612	0.15452	0.2657	9.74	19.03	23.49	32.78
Wdr4	115.22	126.66	146.18	148.54	0.14345	0.27899	0.47111	0.21489	30.96	21.88	11.44	2.36
Wdr6	154.79	159.57	92.77	117.53	0.13695	0.45901	0.21332	0.03492	-62.02	-42.05	4.78	24.76
Yrdc	171.49	137.899	92.0617	185.071	0.00533	0.07057	0.09653	0.23695	-79.43	47.17	-33.60	93.01

Supplemental Figure Legends

Figure S1. Transcripts regulated in *Alkbh8^{Def}* Saline vs. WT Saline mice in the 6 hour APAP experiment. (A) Enhanced volcano plots were generated for *Alkbh8^{Def}* saline verse WT saline and **(B)** Metascape analysis were performed for $log_2FC > 2.0$ and transcripts regulated were identified.

Figure S2. Selenoproteins measured after 6 hour APAP exposure. GPX4, TRXR1 and SELS protein levels in the liver were evaluated using the ProteinSimple WES system. Statistical significance of biological replicates (N = 3) was measured by an unpaired t-test with ($^*p < 0.05$, $^{**}p < 0.01$, $^{***} p < 0.001$).

Figure S3. Remainder of measured epitranscriptomic marks in mouse liver tissue after APAP (6 hours). WT and *Alkbh8^{Def}* mice (N = 3) were exposed to a single 600 mg/kg dose of APAP and livers were harvested 6 hours after dosing. Modifications were measured using LC-MS/MS. Statistical significance of biological replicates (N = 3) was measured by an unpaired t-test with ($^*p < 0.05$, $^{**}p < 0.01$, $^{***} p < 0.001$).

Figure S4. Transcripts regulated in *Alkbh8^{Def}* Saline verse WT Saline mice in the 4 day APAP experiment. (A) Enhanced volcano plots were generated for *Alkbh8^{Def}* saline verse WT saline and **(B)** Metascape analysis were performed for $log_2FC > 2.0$ and transcripts regulated were identified.

Figure S5. Transcripts regulated in *Alkbh8^{Def}* APAP verse WT APAP in the 4 day experiment. Wildtype and *Alkbh8^{Def}* mice (N = 3) were exposed to daily doses of 600 mg/kg APAP over 4 days and tissue was harvested 24 hours after the fourth dose was administered and RNA was purified for analysis by mRNA-seq. **(A)** Enhanced volcano plots were generated for

Alkbh8^{Def} APAP verse WT APAP and **(B)** Metascape analysis were performed for $log_2FC > 2.0$ and transcripts regulated were identified.

Figure S6. Selenoprotein S expression elevated in *Alkbh8^{Def}* liver tissue after 4 day APAP exposure. SELS expression was evaluated using the ProteinSimple WES system. Statistical significance of biological replicates (N = 3) was determined using an unpaired t-test with ($^{*}p < 0.05$, $^{**}p < 0.01$, $^{***}p < 0.001$).

Figure S7. tRNA modifications measured after 4 day APAP exposure. (A) WT and *Alkbh8^{Def}* mice (N=3) were exposed to a daily 600 mg/kg dose of APAP and livers were harvested 4 days after dosing. **(B)** Individual bar graphs showing average concentrations of various tRNA chemical modifications measured using LC-MS/MS. Statistical significance of biological replicates (N = 3) were measured by an unpaired t-test with ($^{*}p < 0.05$, $^{**}p < 0.01$, $^{***}p < 0.001$).

Supplemental Figure S1.

A)

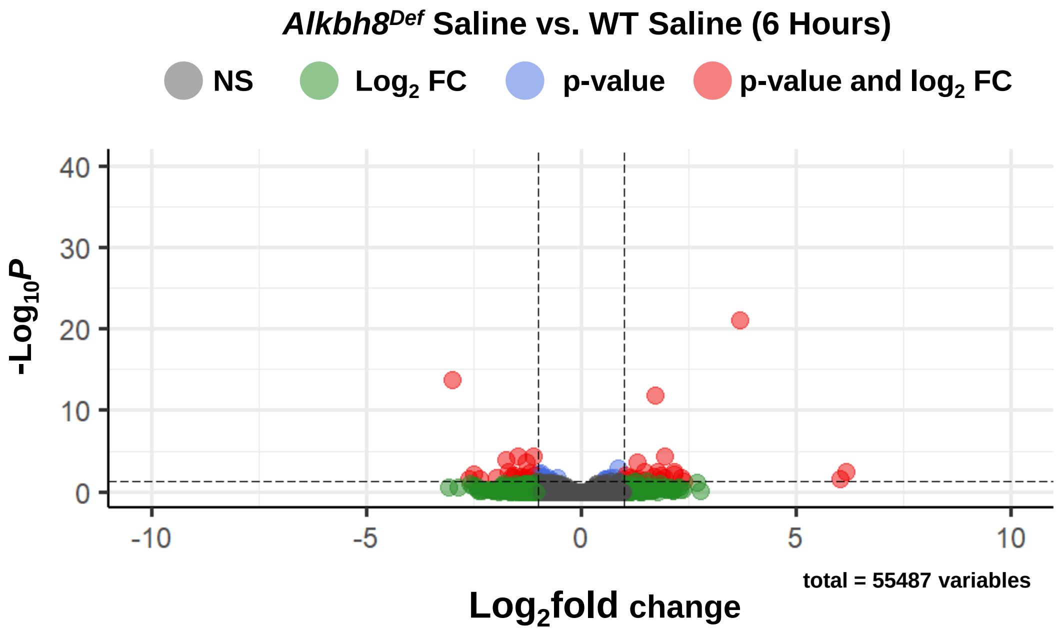

B)

Upregulated

Ensembl ID	Gene Symbol	Description	Biological Process (GO)
ENSMUSG00000038754	**Elovl3**	elongation of very long chain fatty acids (FEN1/Elo2, SUR4/Elo3, yeast)-like 3	GO:0019367 fatty acid elongation, saturated fatty acid;GO:0019368 fatty acid elongation, unsaturated fatty acid;GO:0034625 fatty acid elongation, monounsaturated fatty acid
ENSMUSG00000025229	**Pitx3**	paired-like homeodomain transcription factor 3	GO:1904935 positive regulation of cell proliferation in midbrain;GO:1904933 regulation of cell proliferation in midbrain;GO:0033278 cell proliferation in midbrain
ENSMUSG00000037583	**Nr0b2**	nuclear receptor subfamily 0, group B, member 2	GO:0032922 circadian regulation of gene expression;GO:0032024 positive regulation of insulin secretion;GO:0090277 positive regulation of peptide hormone secretion
ENSMUSG00000044646	**Zbtb7c**	zinc finger and BTB domain containing 7C	GO:1903025 regulation of RNA polymerase II regulatory region sequence-specific DNA binding;GO:2000677 regulation of transcription regulatory region DNA binding;GO:0045600 positive regulation of fat cell differentiation
ENSMUSG00000026077	**Npas2**	neuronal PAS domain protein 2	GO:1903367 positive regulation of fear response;GO:2000987 positive regulation of behavioral fear response;GO:0051775 response to redox state
ENSMUSG00000038508	**Gdf15**	growth differentiation factor 15	GO:0060400 negative regulation of growth hormone receptor signaling pathway;GO:0060398 regulation of growth hormone receptor signaling pathway;GO:0002023 reduction of food intake in response to dietary excess

Downregulated

Ensembl ID	Gene Symbol	Description	Biological Process (GO)
ENSMUSG00000066687	**Zbtb16**	zinc finger and BTB domain containing 16	GO:0048133 male germ-line stem cell asymmetric division;GO:0051138 positive regulation of NK T cell differentiation;GO:0042078 germ-line stem cell division
ENSMUSG00000048794	**Cfap100**	cilia and flagella associated protein 100	GO:0008150 biological_process
ENSMUSG00000091898	**Tnnc1**	troponin C, cardiac/slow skeletal	GO:0032972 regulation of muscle filament sliding speed;GO:0002086 diaphragm contraction;GO:0003011 involuntary skeletal muscle contraction
ENSMUSG00000024365	**Cyp21a1**	cytochrome P450, family 21, subfamily a, polypeptide 1	GO:0006705 mineralocorticoid biosynthetic process;GO:0008212 mineralocorticoid metabolic process;GO:0006704 glucocorticoid biosynthetic process

Supplemental Figure S2.

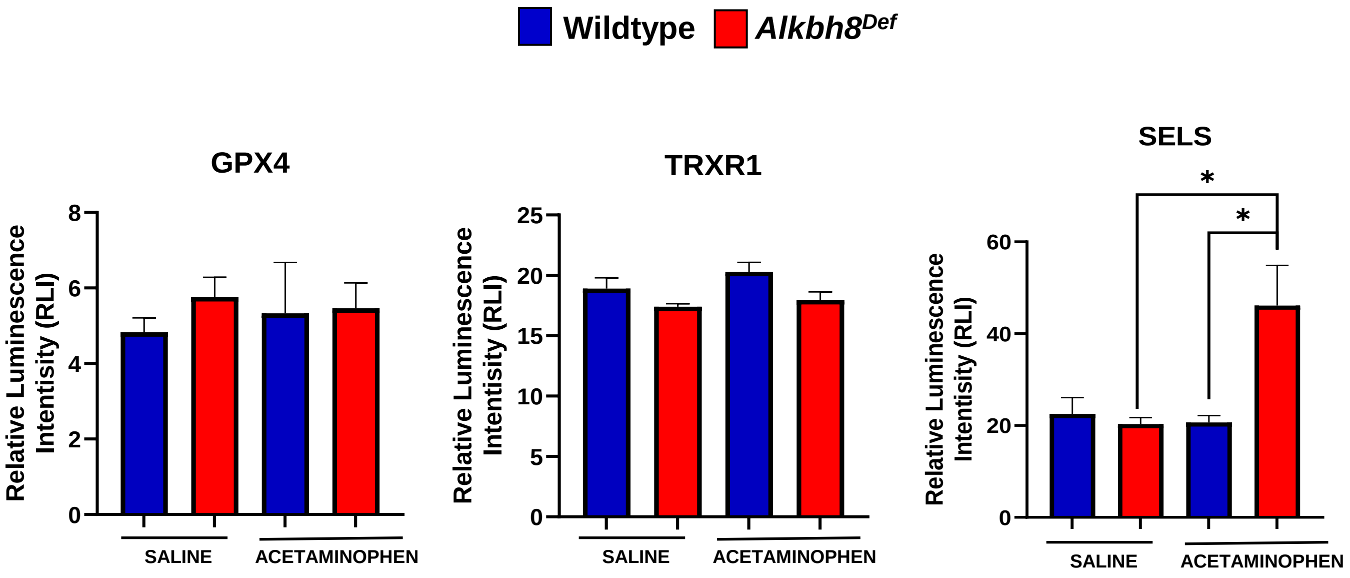

Supplemental Figure S3.

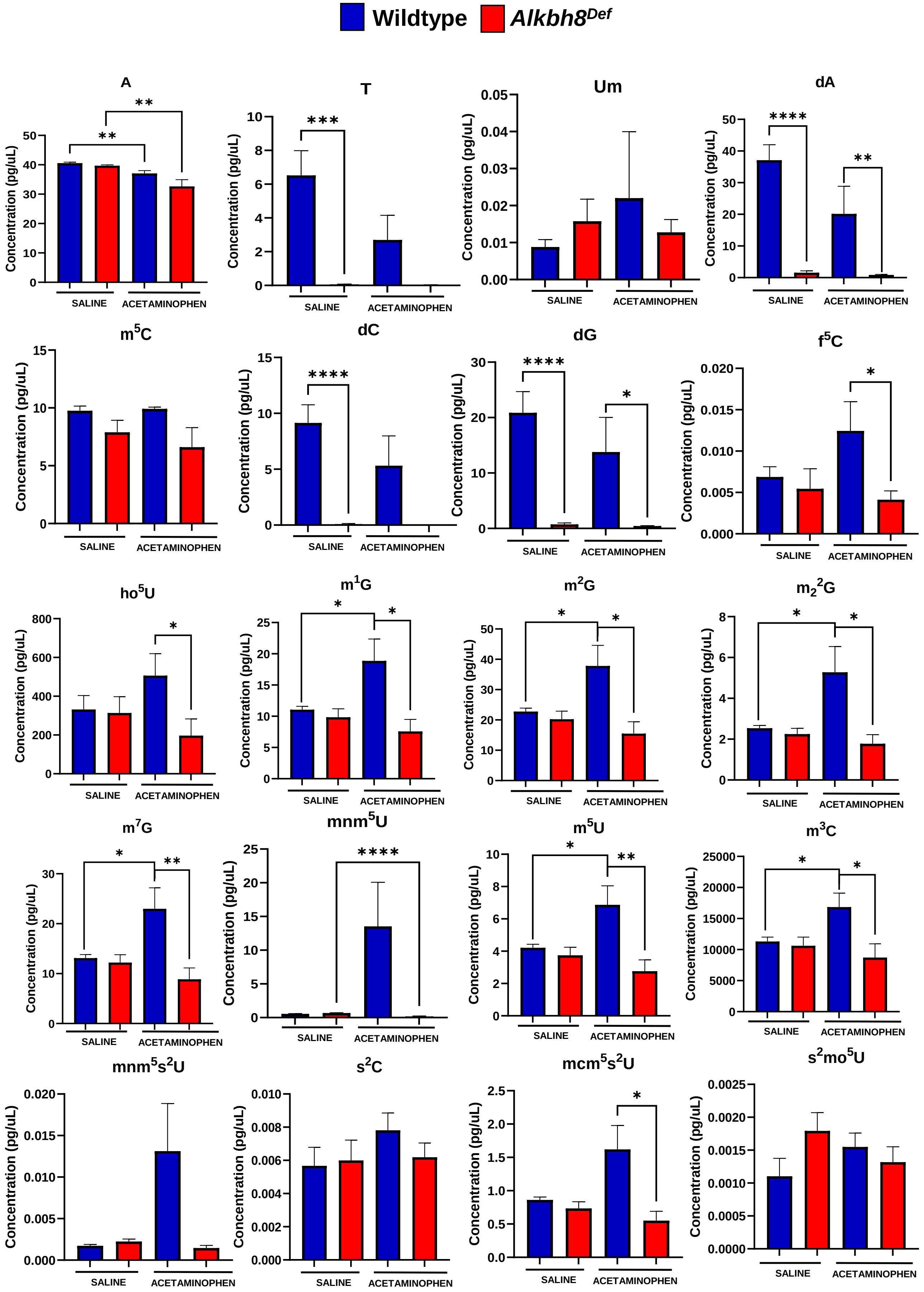

Supplemental Figure S4.

A)

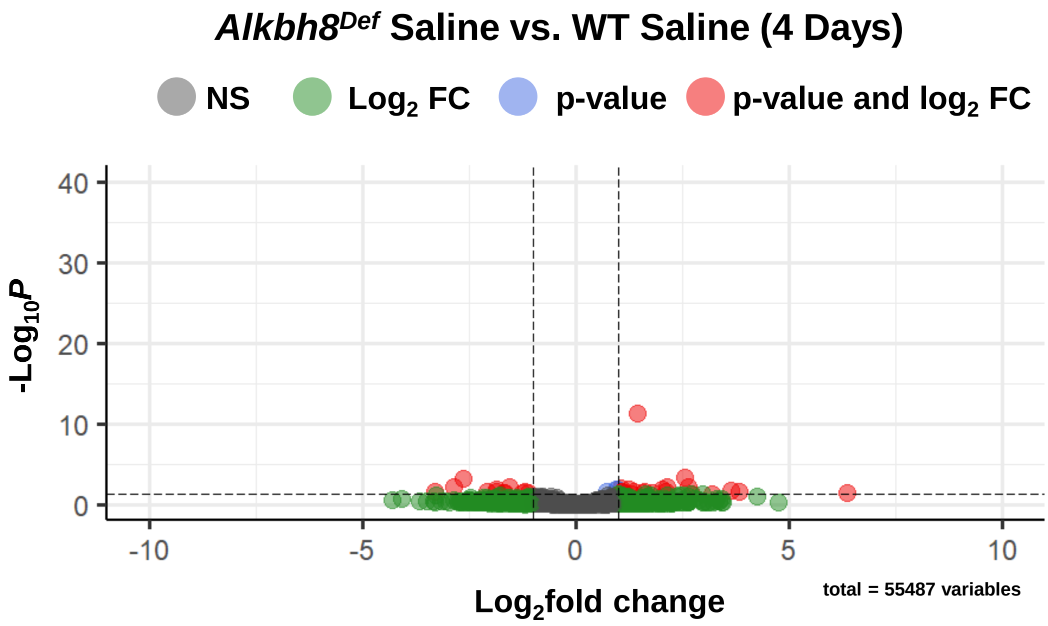

B)

Upregulated

Ensembl ID	Gene Symbol	Description	Biological Process (GO)
ENSMUSG00000035498	**Cdcp1**	CUB domain containing protein 1	GO:0008150 biological_process
ENSMUSG00000022351	**Sqle**	squalene epoxidase	GO:0140042 lipid droplet formation;GO:0034389 lipid droplet organization;GO:0016126 sterol biosynthetic process
ENSMUSG00000041782	**Lad1**	ladinin	
ENSMUSG00000025229	**Pitx3**	paired-like homeodomain transcription factor 3	GO:1904935 positive regulation of cell proliferation in midbrain;GO:1904933 regulation of cell proliferation in midbrain;GO:0033278 cell proliferation in midbrain
ENSMUSG00000024036	**Slc37a1**	solute carrier family 37 (glycerol-3-phosphate transporter), member 1	GO:0015712 hexose phosphate transport;GO:0015760 glucose-6-phosphate transport;GO:0035435 phosphate ion transmembrane transport
ENSMUSG00000026077	**Npas2**	neuronal PAS domain protein 2	GO:1903367 positive regulation of fear response;GO:2000987 positive regulation of behavioral fear response;GO:0051775 response to redox state
ENSMUSG00000025185	**Loxl4**	lysyl oxidase-like 4	GO:0018057 peptidyl-lysine oxidation;GO:0018158 protein oxidation;GO:0030199 collagen fibril organization
ENSMUSG00000023067	**Cdkn1a**	cyclin-dependent kinase inhibitor 1A (P21)	GO:1905178 regulation of cardiac muscle tissue regeneration;GO:1905179 negative regulation of cardiac muscle tissue regeneration;GO:0061026 cardiac muscle tissue regeneration

Downregulated

Ensembl ID	Gene Symbol	Description	Biological Process (GO)
ENSMUSG00000059060	**Rad51b**	RAD51 paralog B	GO:0010971 positive regulation of G2/M transition of mitotic cell cycle;GO:1902751 positive regulation of cell cycle G2/M phase transition;GO:0001832 blastocyst growth
ENSMUSG00000038060	**Dlec1**	deleted in lung and esophageal cancer 1	GO:0008285 negative regulation of cell proliferation;GO:0042127 regulation of cell proliferation;GO:0008283 cell proliferation
ENSMUSG00000022528	**Hes1**	hes family bHLH transcription factor 1	GO:0061105 regulation of stomach neuroendocrine cell differentiation;GO:0061106 negative regulation of stomach neuroendocrine cell differentiation;GO:1905933 regulation of cell fate determination

Supplemental Figure S5.

A)

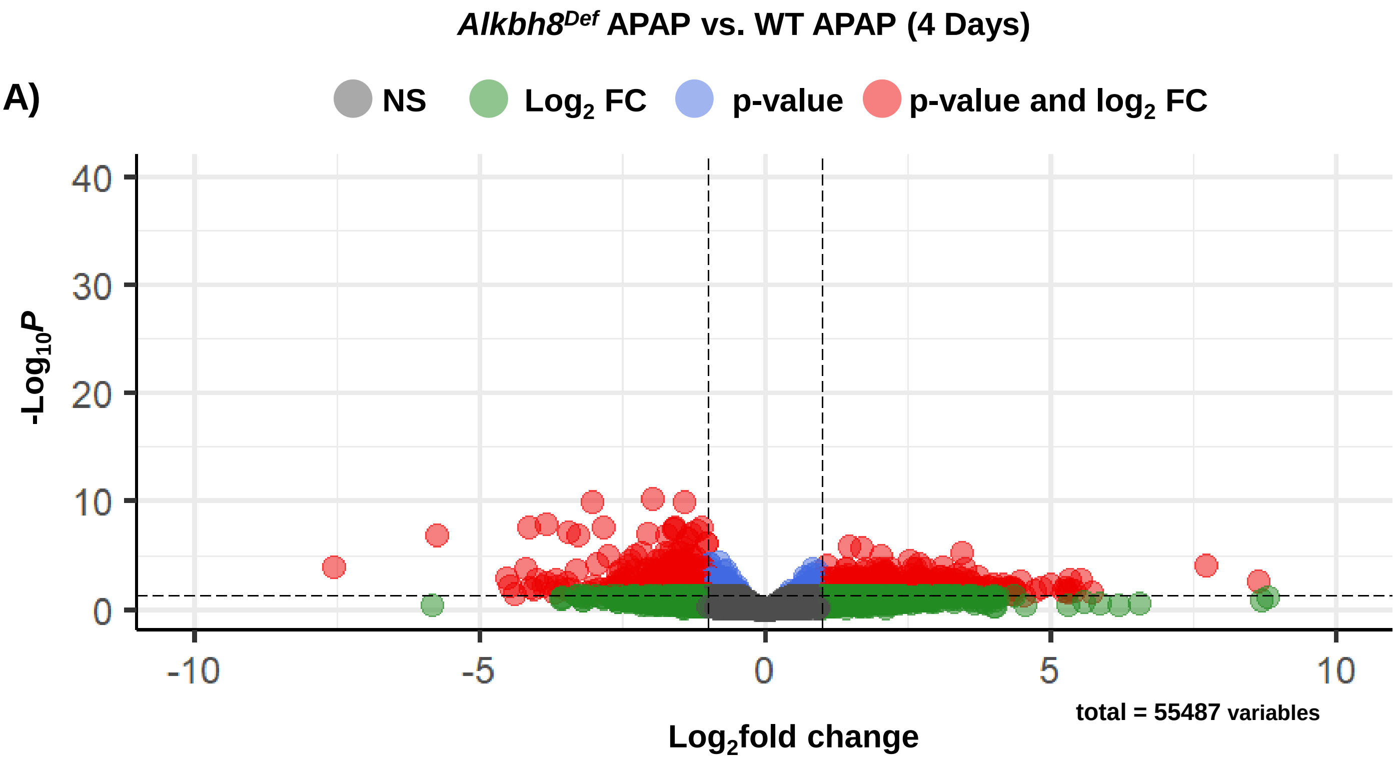

B)

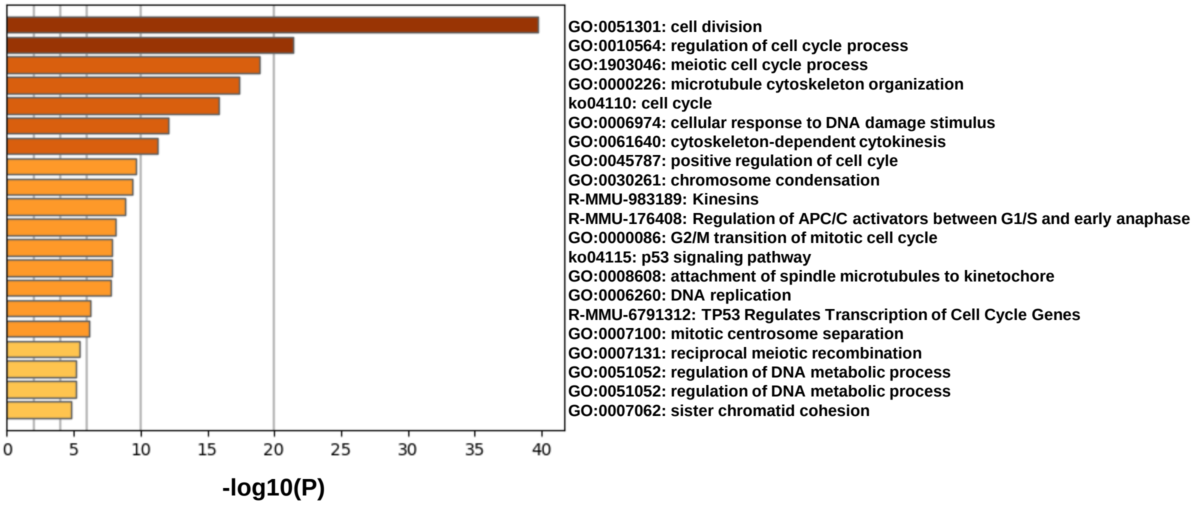

Supplemental Figure S6.

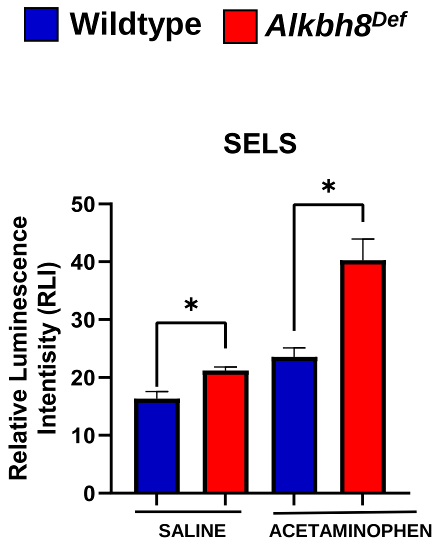

Supplemental Figure S7.

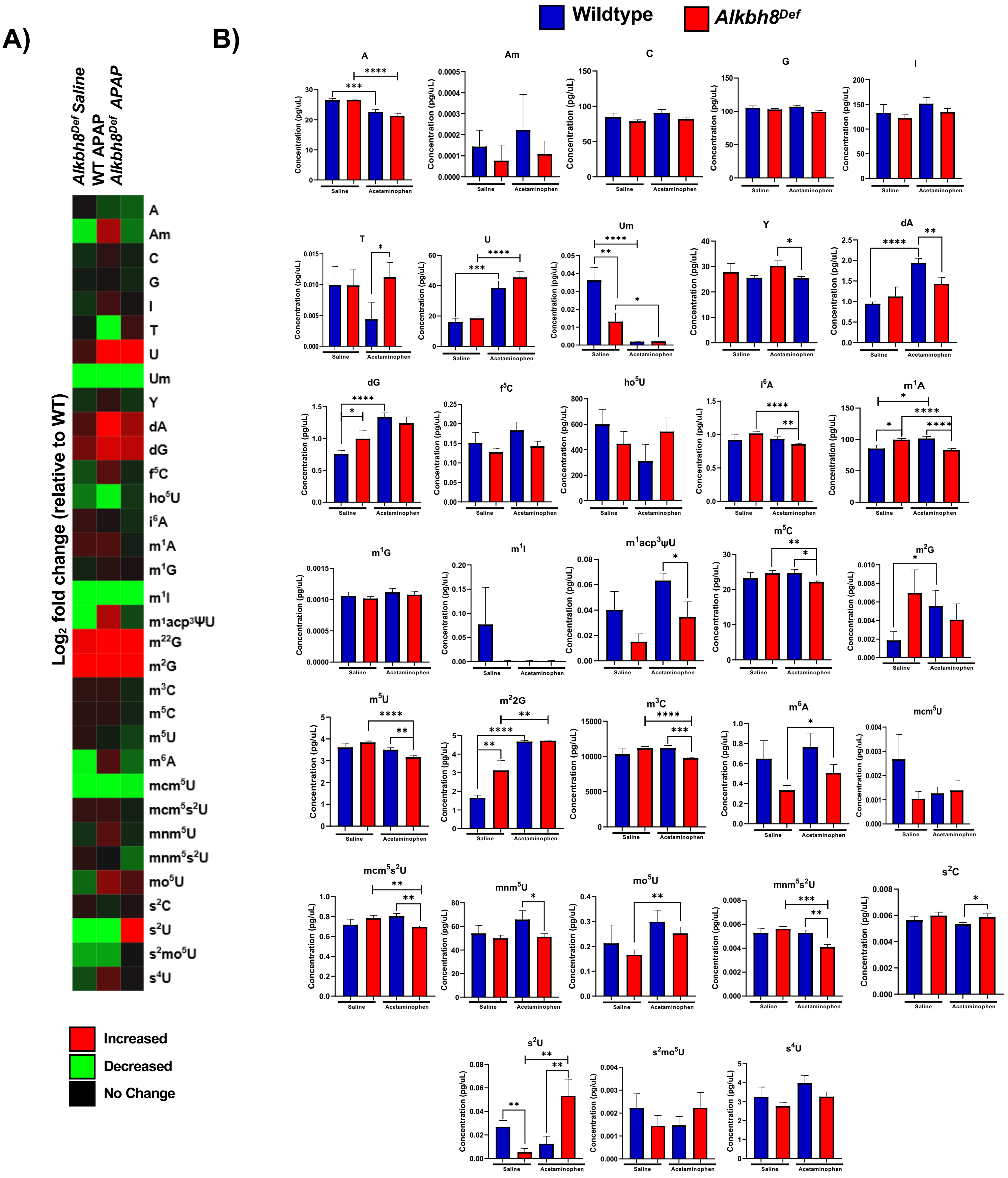

Supplement: Supplementary file 1 [file genes-13-00421-s001.zip › genes-1577549-supplementary.pdf]
